# Higher risk cannabis use indicators among people living in Canada: a cross-sectional study examining the association with socio-demographic and socio-economic factors

**DOI:** 10.1186/s13011-026-00722-9

**Published:** 2026-04-06

**Authors:** Samantha Goodman, Matthew Dann, Hanan Abramovici

**Affiliations:** Office of Cannabis Science and Surveillance, Controlled Substances and Cannabis Branch, Health Canada, 150 Tunney’s Pasture Driveway, Ottawa, ON K1A 0T6 Canada

**Keywords:** Cannabis, Survey, Data disaggregation, Risk behaviours, Canada

## Abstract

**Background:**

In 2018, the *Cannabis Act* regulated the production and sale of cannabis for non-medical purposes in Canada. With the aim of continuing to protect the public health and safety of Canadians as the legal cannabis market matures, we conducted a thorough data disaggregation to identify segments of the cannabis consumer base in Canada who are more likely to engage in ‘higher risk’ cannabis use behaviours. The analysis also aligned with the Canadian federal department of health’s Sex- and Gender-Based Analysis Plus Action Plan.

**Methods:**

Participants were respondents from the 2023 and 2024 cycles of the Canadian Cannabis Survey who reported consuming cannabis in the previous 12 months (*n* = 7,238). Sixteen higher risk cannabis use outcomes were identified, along with 11 socio-demographic/socio-economic characteristics (herein ‘covariates’). Outcomes were arranged in four categories: heavy cannabis consumption; use of higher risk cannabis products; negative effects of cannabis use on one’s life; and threats to personal or public safety. Prevalence of each outcome was estimated among past 12-month consumers. For each outcome, adjusted logistic regression models tested differences between subgroups for each covariate. After Bonferroni correction for multiple comparisons, a threshold of *p* < 0.003 was used.

**Results:**

Over three-quarters (78%) of consumers engaged in at least one higher risk outcome (mean = 2.9, SD = 2.8). After adjustment, models revealed that several subgroups tended to engage in higher risk outcomes. These included younger people, males, those reporting lower education and household income levels, and those who reported having poor/fair mental health. There were also higher rates of certain higher risk outcomes among specific ethnic groups and sexual and gender minorities, but these differences became non-significant in adjusted models.

**Conclusions:**

Study results may be used to identify subgroups of cannabis consumers in Canada who are more likely to engage in higher risk cannabis behaviours and who may benefit from more targeted public education or harm reduction initiatives.

**Supplementary Information:**

The online version contains supplementary material available at 10.1186/s13011-026-00722-9.

## Background

Canada has high rates of cannabis use, with recent estimates from national surveys suggesting that approximately a fifth to a quarter of Canadians had used cannabis in the previous year [1, 2]. In 2001, Canada legalized the possession and personal/designated production of cannabis for medical purposes through the *Marihuana Medical Access Regulations* [3]. In 2015, the federal Liberal Party platform included a promise to “legalize, regulate, and restrict access to marijuana” [4]. The *Cannabis Act*, which legalized and regulated cannabis for non-medical purposes, was passed on October 17, 2018 [5]. Objectives of the *Act* include keeping cannabis out of the hands of youth, restricting the illicit cannabis market, and protecting public health and safety by allowing adults access to legal cannabis [6]. The Canadian Cannabis Survey (CCS) was developed and implemented by Health Canada, Canada’s federal department of health, to understand the impacts of legalization and regulation by obtaining information about the knowledge, attitudes and detailed behaviours of Canadians with respect to cannabis use over time. It was piloted in 2017 prior to legalization and was conducted annually until 2024, when its frequency changed to biennial. CCS examines detailed patterns of cannabis use across the 10 provinces and three territories. This includes quantities of cannabis consumed and use of cannabis for medical and non-medical purposes; attitudes and beliefs related to cannabis; potential problematic use, including through the Severity of Dependence Scale (SDS) [7]; sources of cannabis; and issues of public safety, such as impaired driving and using cannabis at work or school [1].

In 2022, an Expert Panel was tasked with conducting a Legislative Review of the *Cannabis Act*. One of the recommendations in the Panel’s final report was that the Government of Canada should improve its collection and publication of cannabis data that is disaggregated by demographic indicators such as race [8]. In parallel, Health Canada has developed a Sex- and Gender-Based Analysis Plus (SGBA Plus) Action Plan, which aims to strengthen the systematic integration of sex, gender and diversity considerations in its research [9].

Typically, national data on cannabis use is reported by sex and age, as well as by province/territory (PT) for key outcomes [1, 2]. In 2025, Health Canada published interactive data tools for the Canadian Substance Use Survey (CSUS), Canadian Student Alcohol and Drugs Survey and Canadian Postsecondary Education Alcohol and Drugs Survey [10–12]. These tools allow the public to systematically disaggregate cannabis (and other substance) use data by demographic factors. To date, CCS results published on Library and Archives Canada have been disaggregated by sex and three age groups (16–19, 20–24, 25 + years), with comprehensive disaggregation by other demographic variables conducted only for past 12-month cannabis use [13].

Data from CCS suggest that the prevalence of past 12-month cannabis use among individuals aged 16 and older increased immediately between spring 2018 (22%) and spring 2019 (25%) and then plateaued post-legalization in 2020–2024 (25–27%) [1]. However, trends in past 12-month use do not tell the whole story. Past 12-month use represents a myriad of consumption patterns, ranging from using once in the past year to using multiple times per day, which is a known risk factor for various health harms. To address the recommendation of the Expert Panel and the SGBA Plus Action Plan, the present study aimed to conduct a detailed disaggregation of CCS data, with a specific focus on cannabis consumers who engaged in ‘higher risk’ patterns of use. The chosen indicators centred on risks to public health and safety (e.g., driving after cannabis use, purchasing from illicit sources), as well as frequent, heavy use of cannabis (e.g., using cannabis weekly or daily, spending a good portion of the day ‘high’, and using before or during work/school) and consumption of higher potency products. These behaviours have been associated with various harms, including progression to dependence or cannabis use disorder (CUD). CUD is characterized by a continued problematic pattern of cannabis use despite negative consequences, causing significant distress or impaired functioning [14–19].

It is beyond the scope of this paper to detail the state of all higher risk cannabis use outcomes following non-medical legalization in Canada, and indeed, review studies provide a thorough overview [20, 21]. Briefly, there is preliminary research regarding some of the key risk behaviours chosen for this analysis. For one, cannabis-impaired driving was categorized as a threat to public safety, as it has been associated with reduced performance capacity and increased risk of collision [22]. Findings regarding its association with non-medical cannabis legalization are mixed. Surveys, including CCS, show decreases in self-reported driving after cannabis use following legalization, whereas toxicology data and roadside surveys show increases, potentially due to increased roadside surveillance and toxicology testing following legalization [20]. Secondly, purchasing cannabis from an illicit source was categorized as a threat to public health. Unlike cannabis purchased on the legal market in Canada, cannabis purchased from illicit sources is not required to be tested for pesticides, heavy metals or microbial contaminants, and is not subject to mandatory health warning messages^1^ or mandatory labelling of concentrations of tetrahydrocannabinol (THC) – the primary psychoactive component of cannabis [23–25]. As such, use of cannabis from illicit sources may lead to improper dosing (including over-ingestion of THC) or may otherwise pose a risk to one’s health from exposure to harmful contaminants. Purchasing cannabis from illicit sources appears to have decreased substantially following non-medical cannabis legalization, with over 70% of Canadians ‘usually’ obtaining their cannabis from legal sources in 2022–2024 [1, 26, 27]. However, a sizable proportion of Canadians still access the illicit market at least sometimes. Thirdly, several risk behaviours examined herein represent patterns of heavy cannabis use, which may lead to CUD or negative mental health outcomes. Several studies have found increases in CUD and related diagnoses (e.g., schizophrenia, psychosis) as well as cannabis-related hospitalizations since legalization of non-medical cannabis in Canada, while another observed a decrease in CUD [28]. Given that part of the early post-legalization period coincided with the COVID-19 pandemic, associations between legalization and patterns of cannabis use and misuse are difficult to untangle. However, early research also suggests that CUD may be associated with increased risk of adverse physical and mental health outcomes and mortality [29, 30]. This highlights the importance of identifying populations most at risk for these risky patterns of cannabis use, which was the focus of this paper.

A range of factors with which to disaggregate these higher risk outcomes were selected. These included socio-demographic characteristics, socio-economic standing, and considerations related to the respondent’s residence (including community size and provincial/territorial cannabis retail model). The latter is important because under the *Cannabis Act*, PTs are responsible for cannabis distribution and sale. Community size and cannabis retail model are important because they may affect retail density (number of cannabis retail stores per capita) [31], which in turn affects access to cannabis. Retail model also may influence the types of products sold (i.e., by government- versus privately run retailers).

Previous research on subgroup differences in the prevalence and frequency of cannabis use in Canada and the US [1, 10, 27, 32–36] led us to expect that higher risk outcomes would differ across demographic variables. However, the analysis was considered exploratory and specific hypotheses were not formed for particular population subgroups.

## Methods

CCS is an electronic survey of people aged 16 years and older living in Canada, conducted by the survey firm Advanis. Questionnaires are published online in methodological reports [13, 37]. In brief, CCS was substantially revised in 2023 and underwent no major changes in 2024 with the exception of a few added questions, including use of infused pre-rolls (i.e., joints in which dried cannabis is mixed with cannabis extract, which increases its potency) [1].

Estimated prevalence of past 12-month cannabis use was the same in the 2023 and 2024 CCS (26% for both) as were other key indicators such as social acceptability of cannabis use and age of initiation to cannabis use [1, 38]. Given the stability of estimates and alignment of survey measures and sampling methodologies across the two cycles, the 2023 and 2024 samples were pooled for this analysis. This created one dataset representing a 2-year period average. This allowed us to engage in a detailed SGBA Plus analysis without the issue of small cell sizes. Sample weights were adjusted for the pooled analysis by dividing by the number of survey cycles [39, 40]. The sample comprised respondents who consumed cannabis in the previous 12 months. Those who did not provide a response regarding past 12-month use were excluded (*n* = 77), resulting in a sample of 7,238 consumers.

## Measures

### Higher risk outcomes

**Fig. 1:**
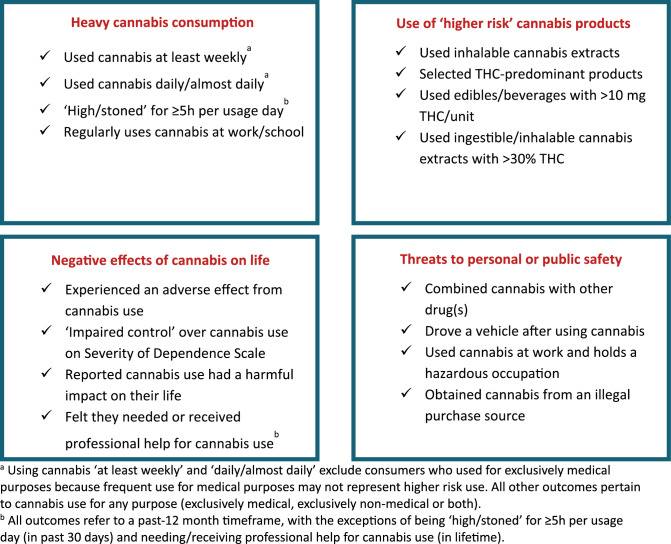
Higher risk cannabis outcomes used in the analysis, arranged by category. **a** Using cannabis ‘at least weekly’ and ‘daily/almost daily’ exclude consumers who used for exclusively medical purposes because frequent use for medical purposes may not represent higher risk use. All other outcomes pertain to cannabis use for any purpose (exclusively medical, exclusively non-medical or both). **b** All outcomes refer to a past-12 month timeframe, with the exceptions of being ‘high/stoned’ for ≥5 h per usage day (in past 30 days) and needing/receiving professional help for cannabis use (in lifetime)

Sixteen higher risk cannabis use outcomes were identified and arranged into four categories, with four outcomes each. As shown in Fig. 1, these were heavy cannabis consumption; use of higher risk cannabis products; negative effects of cannabis use on one’s life; and threats to personal or public safety. The operational definition of each outcome is detailed in Table 1 and its accompanying footnotes.

**Table 1:** Prevalence of ‘higher risk’ cannabis use outcomes among past 12-month cannabis consumers in the Canadian Cannabis Survey 2023–2024 (*n* = 7,238), % (n)

Variable	2023 (*n* = 3,687)	2024 (*n* = 3,551)	Total (*n* = 7,238)	Difference between cycles (*p*-value), Pearson’s *X*^2^ test
**Heavy cannabis consumption**				
At least weekly cannabis use, among those who used for non-medical purposes in past 12 months (*n* = 6,198)	43.3% (1,399)	43.7% (1,340)	43.5%^↑^ (2,739)	0.743
Daily/almost daily cannabis use, among those who used for non-medical purposes in past 12 months (*n* = 6,198)	23.0% (736)	23.7% (730)	23.4% (1,466)	0.567
‘High/stoned’ for ≥ 5 h per day in the past 30 days, among those who used cannabis in the past 30 days (*n* = 4,678)	15.1% (366)	18.3% (409)	16.7% (775)	0.008
Sometimes/often/always used cannabis within 2 h of or during work/school in past 12 months (*n* = 7,186)	5.6% (218)	7.7% (276)	6.9% (494)	**0.0007**
**Use of higher risk cannabis products**				
Used inhalable cannabis extracts in past 12 months ^a^ (*n* = 6,764)	42.0% (1,519)	47.1% (1,596)	44.5% (3,115)	**< 0.0001**
Usually selects THC-only or THC-predominant cannabis products ^b^ (*n* = 7,208)	35.3% (1,330)	34.5%^↑^ (1,234)	34.9% (2,564)	0.553
Usually consumes cannabis with potency of > 30% THC, among those who use inhaled/ingestible extracts ^c^ (*n* = 1,405)	54.4% (340)	45.4% (380)	49.0% (720)	**< 0.003**
Usually consumes edibles/beverages with > 10 mg THC per piece/beverage, among edible/beverage consumers ^d^ (*n* = 2,097)	23.2% (263)	27.2% (285)	25.3% (548)	0.061
**Negative effects of cannabis on personal life**				
Experienced an adverse or negative health effect from cannabis use in past 12 months ^e^ (*n* = 6,879)	30.0% (1,087)	30.2% (1,035)	30.1% (2,122)	0.816
Cannabis had a somewhat or very harmful impact on life in past 12 months ^f^ (*n* = 7,180)	18.1% (681)	20.0% (692)	19.1% (1,373)	0.066
Classified as having ‘impaired control’ over one’s cannabis use in past 12 months (score ≥ 4) on Severity of Dependence Scale ^g^ (*n* = 7,069)	11.0% (403)	12.3% (412)	11.6% (815)	0.129
Felt they needed or received professional help for cannabis use in lifetime, among those who used cannabis more than once in lifetime (*n* = 6,892)	6.0% (204)	6.7% (224)	6.4% (428)	0.324
**Threats to personal or public safety**				
Often or always combined cannabis with other drug(s) in past 12 months ^h^ (*n* = 7,238)	28.9% (1,071)	31.1% (1,125)	30.0% (2,196)	0.072
Drove within 2 h of smoking/vaping cannabis or 4 h of ingesting cannabis edibles in past 12 months (*n* = 7,033)	15.3% (564)	18.4% (646)	16.8% (1,210)	**0.002**
Used cannabis at/within 2 h of work in past 12 months and has a hazardous occupation, among those employed in the past 12 months ^i^ (*n* = 6,838)	4.8% (180)	5.9% (199)	5.4% (379)	0.076
Usually purchased cannabis from an illegal store, illegal website or dealer in past 12 months (*n* = 7,121)	3.4% (123)	3.3% (120)	3.3% (243)	0.822

Significant differences at *p* < 0.003 are bolded

↓Indicates number should be rounded down if reported as a whole number

↑Indicates number should be rounded up if reported as a whole number

^a^Use of cannabis vape pens/cartridges; hash/kief; concentrates/extracts (e.g., wax, shatter, budder, butane honey oil); or concentrate-infused pre-rolls (in 2024 only) in past 12 months

^b^Respondent selected ‘Higher THC, lower CBD’ or ‘THC only’ when asked which cannabis products they typically choose

^c^Among respondents who had consumed hash/kief, vape pens/cartridges, concentrates/extracts or oils for oral use in the past 12 months and entered a usual THC concentration for their products in %THC or mg/g THC. Excludes those who selected ‘Don’t know’ or who entered a THC concentration in mg (because product weight/volume was unknown)

^d^Among respondents who had consumed cannabis edibles or beverages in the past 12 months and entered a usual THC concentration for their products in mg THC per unit. Excludes those who selected ‘Don’t know’

^e^Includes any of the following: nausea/vomiting; heart or blood pressure problems; feeling faint/passing out/loss of consciousness; anxiety/panic attack/rapid heartbeat; hallucinations/psychosis/flashbacks; dissociation or depersonalization; slowed breathing/lung problems; allergic reaction; confusion/disorientation; unusual behaviour (e.g., agitation, slurred speech); chest pain/discomfort; loss of coordination/unsteadiness/vertigo; headache; diarrhea; seizure; drowsiness/lethargy; muscle weakness; other negative effect

^f^Includes impact on any of the following: friendships or social life; physical health; mental health; home life or marriage; performance at work or school; and quality of life

^g^Defined as a score of ≥ 4 on the Severity of Dependence Scale, which asks five items about one’s cannabis use in the past 12 months

^h^‘Combined’ was defined as being mixed or consumed at the same time. Drugs included any of the following: alcohol; tobacco; e-cigarettes with nicotine; opioids; stimulants; sedatives/tranquilizers; and hallucinogens/dissociatives

^i^Hazardous occupations included any of the following: driving a motor vehicle; operating equipment, machinery or tools; working from heights; working with hazardous chemicals, flammable liquids or gases; sharps work; working near hot surfaces, open flames or steam; electrical work; handling loads > 20 kg; working near flying particles or falling objects; and being responsible for the care/wellbeing of others

When interpreting results related to usual purchase source, readers should also note that many youth in the sample were below the minimum legal age (MLA) for cannabis. Presently, the provinces of Alberta and Quebec have MLAs for possession and purchasing of cannabis of 18 and 21 years, respectively, and the remaining 11 provinces/territories have a MLA of 19 years.

Also note that two measures of frequent cannabis use were included in the ‘heavy cannabis consumption’ category to explore whether patterns of frequent cannabis use in socio-demographic subgroups differed between those who used ‘at least weekly’ (which might represent cannabis use in a social setting; for example, on weekends with friends) versus ‘daily/almost daily’ (which might represent solitary cannabis use and is associated with cannabis dependence) [41]. The sample for these two outcomes excluded consumers who used for exclusively medical purposes because frequent medical use may not necessarily represent higher risk use. For example, daily cannabis use may be recommended by a medical professional, or medical use may primarily constitute use of cannabidiol (CBD)-predominant products, which are weakly^2^ or not at all psychoactive. All other outcomes pertain to cannabis use for any purpose (exclusively medical, exclusively non-medical, or both). Use of cannabis during pregnancy and/or breastfeeding was considered as an outcome but was excluded due to insufficient sample (*n* = 56, 0.8% of sample).

### Coding of behavioural outcome variables

For the purpose of this analysis, ‘inhalable cannabis extracts’ were defined as vape pens/cartridges, hash/kief, concentrates (e.g., wax, shatter, budder), and infused pre-rolls (2024 survey only). These products tend to carry higher health risks due to their high THC levels [14, 16].

For several variables, it was necessary to select a threshold for statistical analysis. Decisions were made based on logical cut-points, existing literature/regulations, and data distributions. Consumers were asked to report how many hours they had been ‘high/stoned’ on a typical usage day in the past 30 days. Because the data revealed that > 80% of consumers selected response options below 5 h, > 5 h was chosen to reflect heavy use. Respondents who had consumed each product in the past 12 months were asked to enter the usual THC levels of their products. Since Canadian regulations cap legal cannabis edibles at 10 mg per package [42], > 10 mg/unit was selected to signify use of higher potency edibles/beverages.^3^ For use of higher potency inhalable/ingestible extracts (vape pens/cartridges, hash/kief, concentrates, and oils for oral use), > 30% THC was chosen because this represents a plausible potency limit for dried flower/leaf [43], which was excluded from this variable. To measure ‘impaired control’ (also known as psychological dependence) over one’s cannabis use, a score of ≥ 4 on the SDS was used as per previous literature [7]. Finally, for combining cannabis with other drugs, response options of ‘rarely’, ‘sometimes’, ‘often’, and ‘always’ in the past 12 months could reflect co-use of cannabis and other substances. Responses of ‘often’ and ‘always’ were selected to reflect more frequent (i.e., riskier) patterns of substance co-use.

### Covariates

Each outcome was disaggregated by the following 11 socio-demographic/socio-economic characteristics (herein ‘covariates’): **Age group** (16–19 **/** 20–24 **/** 25–34 **/** 35–44 **/** 45–54 **/** ≥55 years)^4^; **Sex at birth** (Male **/** Female); **Gender modality**^5^ (Cisgender **/** Gender diverse); **Sexual orientation** (Heterosexual **/** Gay or Lesbian **/** Bisexual or Another sexual orientation **/** Prefer not to say); **Ethnic group** (White [exclusively selected] **/** Indigenous [non-exclusive] **/** All other ethnic groups [including more than one ethnicity and prefer not to say]); **Highest level of education** (High school or less **/** Trades, college, non-university diploma **/** At least some university); **Household income** (<$50,000 **/** $50,000 to $99,999 **/** ≥$100,000); **Self-rated mental health** (Poor or Fair **/** Other response^6^); **Community size** (Rural/small [< 30,000 people] **/** Medium [30,000 to < 100,000 people] **/** Large [≥ 100,000 people]); **Immigration status** (Born in Canada **/** Born elsewhere); and **Provincial/territorial cannabis retail model**^7^ (Hybrid [public and private] sales **/** Exclusively private sales **/** Exclusively public [i.e., government-run] sales). For several other variables, original response options were collapsed to allow for subgroup analysis.

### Statistical analysis

Descriptive statistics estimated prevalence of each outcome among consumers; respondents who did not answer the question were excluded from the denominator. Pearson’s chi-square tested differences in prevalence of each outcome between 2023 and 2024. For each of the 16 outcomes, unadjusted regression models were conducted to test the effect of each covariate separately (data not shown), followed by adjusted models to test the additive effect of all covariates. Respondents missing data for covariates were excluded using listwise deletion. Adjusted odds ratios and 95% confidence intervals are shown. To account for multiple comparisons (16 regression models), a Bonferroni correction was applied, resulting in a threshold of *p* < 0.003 for significance. Analyses were conducted on weighted estimates using survey procedures in STATA v.17. Further details on weighting and sampling procedures are available in the survey’s methodological reports [13, 37].

## Results

Sample characteristics are summarized in Additional File 1. Just under half the sample (48.4%) was female and approximately three-quarters (76.9%) identified as White.

The prevalence of each higher risk cannabis use outcome is shown in Table 1. Only four outcomes differed significantly between 2023 and 2024, one of which was use of inhalable cannabis extracts. This likely increased due to the addition of a question on infused pre-rolls in 2024.

Over three-quarters (78.4%) of consumers endorsed at least one higher risk outcome, and none endorsed all 16. Consumers endorsed 2.9 outcomes on average (SD = 2.8; range = 0–15). Few (12.6%) consumers endorsed an outcome from all four categories; results for each category are summarized below.

After adjustment for covariates, regression models revealed several subgroups that tended to have a greater likelihood of higher risk outcomes. These included younger people; males; consumers with lower education levels, lower household income brackets, and poor/fair mental health; and consumers born in Canada. Prevalence estimates and regression results for a key outcome within each of the four categories are presented in Tables 2, 3, 4 and 5. Results for remaining outcomes are shown in Additional File 2.

**Table 2:** Prevalence of at least weekly cannabis use among respondents who used cannabis for non-medical purposes in the past 12 months, 2023–2024 Canadian Cannabis Survey (*n* = 6,198)

Covariate	Prevalence (%) (95% CI)	Adjusted Odds Ratio, (95% CI)	*p*-value
Overall	43.5 (42.2–44.9)	F(23, 6588) = 10.57	**< 0.0001**
**Age group (years)**		F(5, 6606) = 6.74	**< 0.0001**
16–19	40.2 (36.3–44.3)	--ref--	--ref--
20–24	46.1 (43.3–49.0)	1.59 (1.24–2.04)	**< 0.0001**
25–34	43.1 (39.9–46.4)	1.86 (1.42–2.43)	**< 0.0001**
35–44	46.9 (43.7–50.0)	2.14 (1.64–2.80)	**< 0.0001**
45–54	39.5^↑^ (36.1–43.1)	1.64 (1.24–2.17)	**< 0.0001**
55 and older	42.9 (39.9–46.0)	1.89 (1.44–2.47)	**< 0.0001**
**Sex**		F(1, 6610) = 46.71	**< 0.0001**
Female	38.7 (36.7–40.7)	--ref--	--ref--
Male	47.7 (45.8–49.6)	1.57 (1.38–1.78)	**< 0.0001**
**Gender modality**		F(1, 6610) = 0.63	0.428
Cisgender	43.3 (41.8–44.7)	--ref--	--ref--
Gender diverse	47.9 (42.0-53.9)	0.88 (0.64–1.21)	0.428
**Sexual orientation**		F(4, 6607) = 1.19	0.313
Heterosexual (straight)	42.9 (41.3–44.4)	--ref--	--ref--
Homosexual (lesbian or gay)	43.0 (36.4–49.9)	0.92 (0.67–1.27)	0.624
Bisexual	47.3 (43.7–50.9)	1.24 (1.00-1.53)	0.053
Other sexual identity	47.3 (43.7–50.9)	1.13 (0.75–1.69)	0.556
Unstated	42.2 (34.0-50.8)	0.92 (0.58–1.45)	0.714
**Ethnic group**		F(2,6609) = 0.97	0.378
White (exclusive category)	43.4 (41.8–44.9)	--ref--	--ref--
Indigenous	52.5^↑^ (46.1–58.8)	1.22 (0.90–1.65)	0.208
Other ethnic group/unstated	42.1 (38.8–45.4)	1.07 (0.89–1.28)	0.487
**Highest education level**		F(2, 6609) = 32.43	**< 0.0001**
High school or less	51.9 (49.5–54.3)	1.94 (1.63–2.30)	**< 0.0001**
Trades/college or non-university diploma or certificate	48.2 (45.5–50.8)	1.59 (1.36–1.86)	**< 0.0001**
At least some university	34.1 (31.9–36.3)	--ref--	--ref--
**Household income**		F(2, 6609) = 9.49	**0.0001**
Less than $50,000	48.9 (45.8–52.1)	1.35 (1.13–1.60)	**0.001**
$50,000-$99,999	47.2 (44.6–49.8)	1.33 (1.15–1.54)	**< 0.0001**
$100,000 or more	38.0 (35.9–40.1)	--ref--	--ref--
**Mental health status**		F(1, 6610) = 16.61	**< 0.0001**
Fair or Poor	50.9 (48.3–53.5)	1.36 (1.17–1.58)	**< 0.0001**
Other response	40.6 (39.0-42.2)	--ref--	--ref--
**Community size**		F(2, 6609) = 1.96	0.142
Rural/small community (< 30,000)	44.5^↑^ (41.9–47.1)	--ref--	--ref--
Medium community (30,000–99,999)	46.1 (43.0-49.3)	1.20 (1.00-1.43)	0.049
Large community (100,000 or more)	42.0 (40.1–43.9)	1.07 (0.92–1.24)	0.391
**Immigration status**		F(1, 6610) = 15.24	**< 0.0001**
Born outside of Canada	34.4 (30.8–38.3)	--ref--	--ref--
Born in Canada	44.9 (43.4–46.4)	1.52 (1.23–1.87)	**< 0.0001**
**Provincial/territorial cannabis retail model**		F(2, 6609) = 8.46	**0.0002**
Hybrid (public & hybrid sales)	44.2 (42.4–46.1)	1.32 (1.13–1.54)	**< 0.0001**
Private sales	47.5^↓^ (44.6–50.3)	1.42 (1.18–1.70)	**< 0.0001**
Public (government-run) sales	37.4 (34.7–40.3)	--ref--	--ref--

Significant differences at *p* < 0.003 are bolded

↑Indicates value should be rounded up if rounded to a whole number

↓Indicates value should be rounded down if rounded to a whole number

Ref=reference group

### Heavy cannabis use

Items in the heavy cannabis use category were endorsed by approximately 4 in 10 consumers (41.3%) overall. As shown in Table 1, frequent cannabis use was the single most common outcome endorsed within this category, with over 4 in 10 respondents who had consumed cannabis for non-medical purposes (43.5%) reporting at least weekly use and almost a quarter reporting the more conservative measure of daily/almost daily use. As shown in Table 2, male consumers were approximately 1.6 times more likely than females to use cannabis at least weekly. Despite most outcomes being more common in youth, all older age groups were more likely than youth aged 16–19 to use cannabis at least weekly, with adults aged 35–44 being more than twice as likely. In addition, consumers living in PTs with exclusively private or hybrid (private and public) cannabis sales were more likely to use at least weekly than those living in PTs with exclusively public sales. The general pattern of results was similar for daily/almost daily use, except that the differences between subgroups were less pronounced (Additional File 2).

Approximately 1 in 6 consumers (16.7%) who had used cannabis in the past 30 days reported being high/stoned for at least 5 h per usage day. This pattern of use was more common among males and those with lower income levels and poor/fair mental health. Regularly consuming cannabis at one’s place of work or school was less prevalent overall (6.6%). This behaviour was more common among 16-19-year-olds (vs. 55 years and older), males, those with a high school education or less, and those with poor/fair mental health (Additional File 2).

### Use of higher risk products

Use of higher risk products was endorsed by the most consumers overall (57.7%). As shown in Table 3, approximately 45% of consumers reported using an inhalable cannabis extract in the past year, and this decreased with age. Compared to adults aged 55 and older, younger people aged 16–19 and 20–24 years were over four and three times more likely, respectively, to report using these products. In addition, consumers living in PTs with exclusively private cannabis sales or a hybrid sales model were significantly more likely to use inhalable cannabis extracts than those living in PTs with public sales.

**Table 3:** Used inhalable cannabis extracts ^a^ in the past 12 months, among past 12-month cannabis consumers, 2023–2024 Canadian Cannabis Survey (*n* = 6,764)

Covariate	Prevalence (%) (95% CI)	Adjusted Odds Ratio, (95% CI)	*p*-value
Overall	44.5 (43.2–45.9)	F(23, 6082) = 22.22	**< 0.0001**
**Age group (years)**		F(5, 6100) = 37.68	**< 0.0001**
16–19	70.2 (66.2–73.9)	4.58 (3.46–6.05)	**< 0.0001**
20–24	60.4 (57.5–63.1)	3.27 (2.69–3.99)	**< 0.0001**
25–34	48.8 (45.6–52.1)	2.43 (2.00-2.95)	**< 0.0001**
35–44	45.0 (42.0-48.1)	2.04 (1.69–2.45)	**< 0.0001**
45–54	39.4 (36.1–42.8)	1.67 (1.37–2.04)	**< 0.0001**
55 and older	28.0 (25.7–30.5)	--ref--	--ref--
**Sex**		F(1, 6104) = 41.74	**< 0.0001**
Female	40.9 (39.0-42.8)	--ref--	--ref--
Male	47.9 (46.1–49.8)	1.51 (1.34–1.72)	**< 0.0001**
**Gender modality**		F(1, 6104) = 1.72	0.189
Cisgender	44.0 (42.7–45.4)	--ref--	--ref--
Gender diverse	53.8 (48.0-59.5)	0.81 (0.59–1.11)	0.189
**Sexual orientation**		F(4, 6101) = 1.39	0.234
Heterosexual (straight)	42.5^↓^ (41.0–44.0)	--ref--	--ref--
Homosexual (lesbian or gay)	44.3 (37.9–50.8)	0.80 (0.59–1.08)	0.147
Bisexual	56.2 (52.6–59.7)	1.11 (0.90–1.38)	0.338
Other sexual identity	45.0 (37.1–53.1)	1.20 (0.81–1.78)	0.370
Unstated		1.31 (0.84–2.02)	0.232
**Ethnic group**		F(2, 6103) = 2.68	0.069
White (exclusive category)	43.4 (42.0-44.9)	--ref--	--ref--
Indigenous	59.2 (53.2–64.9)	1.40 (1.05–1.87)	0.021
Other ethnic group/unstated	45.5^↓^ (42.3–48.7)	1.01 (0.85–1.21)	0.907
**Highest education level**		F(2, 6103) = 29.55	**< 0.0001**
High school or less	56.7 (54.3–59.9)	1.72 (1.46–2.03)	**< 0.0001**
Trades/college or non-university diploma or certificate	46.4 (44.0-48.9)	1.67 (1.44–1.95)	**< 0.0001**
At least some university	34.3 (32.3–36.4)	--ref--	--ref--
**Household income**		F(2, 6103) = 3.80	0.022
Less than $50,000	48.8 (45.9–51.7)	1.16 (0.98–1.37)	0.080
$50,000-$99,999	46.4 (44.0-48.9)	1.21 (1.05–1.40)	0.008
$100,000 or more	40.4 (38.4–42.5)	--ref--	--ref--
**Mental health status**		F(1, 6104) = 35.30	**< 0.0001**
Fair or Poor	57.6 (55.2–60.1)	1.54 (1.33–1.77)	**< 0.0001**
Other response	39.3 (37.8–40.9)	--ref--	--ref--
**Community size**		F(2, 6103) = 0.01	0.989
Rural/small community (< 30,000)	45.1 (42.7–47.5)	--ref--	--ref--
Medium community (30,000–99,999)	46.6 (43.6–49.6)	0.99 (0.83–1.18)	0.909
Large community (100,000 or more)	43.6 (41.8–45.5)	1.00 (0.87–1.16)	0.977
**Immigration status**		F(1, 6104) = 10.69	**0.001**
Born outside of Canada	45.9 (44.5–47.3)	--ref--	--ref--
Born in Canada	35.9 (32.4–39.6)	1.40 (1.14–1.71)	**0.001**
**Provincial/territorial cannabis retail model**		F(2, 6103) = 15.09	**< 0.0001**
Hybrid (public & hybrid sales)	44.1 (42.3–45.8)	1.34 (1.15–1.56)	**< 0.0001**
Private sales	51.7 (49.0-54.4)	1.65 (1.38–1.98)	**< 0.0001**
Public (government-run) sales	38.8 (36.1–41.6)	--ref--	--ref--

Significant differences at *p* < 0.003 are bolded

↑Indicates value should be rounded up if rounded to a whole number

↓Indicates value should be rounded down if rounded to a whole number

Ref=reference group

^a^Use of cannabis vape pens/cartridges; hash/kief; concentrates/extracts (e.g., wax, shatter, budder, butane honey oil); or concentrate-infused pre-rolls (added in 2024 survey) in past 12 months

Regarding use of high-potency products, over a third of consumers (34.9%) reported ‘usually’ selecting products that predominantly contained THC (as opposed to predominantly CBD, equal THC and CBD, a mix of product types, or reporting they did not know). In addition, among consumers of who provided THC levels for their usual cannabis products, almost half (49.0%) of inhalable/ingestible extract consumers reported consuming products with > 30% THC, and a quarter of edible/beverage consumers (25.3%) reported consuming products with > 10 mg THC/unit. Regarding subgroup differences, usually selecting THC-predominant products was more common among males and those with lower education levels. The likelihood of using products with > 30% THC was significantly higher among the three youngest age groups compared to adults 55 and older. Specifically, youth 16–19 and young adults 20–24 and 25–33 were nine, six and three times more likely, respectively, with 82.5% of youth reporting that their usual products of these categories had > 30% THC. Interestingly, youth aged 16–19 were approximately twice as likely as young adults aged 20–24 to report consuming edibles/beverages with > 10 mg THC/unit, with almost half (47.5%) of youth who provided THC levels for their usual edible/beverages reporting potencies above 10 mg, compared to 27.7% of young adults aged 20–24 and approximately a fifth to a quarter of consumers in older age groups (Additional File 2).

### Negative effects of cannabis use on one’s life

Negative effects of cannabis use on one’s life were reported by approximately 4 in 10 consumers (39.0%) overall. Thirty percent (30.1%) reported experiencing at least one adverse/negative health effect from cannabis use, just under 1 in 5 (19.1%) reported that cannabis had a ‘somewhat’ or ‘very’ harmful impact on at least one aspect of their life, and 11.6% were classified as having ‘impaired control’ over their cannabis use on the SDS. Few (6.4%) felt they needed or had received professional help for cannabis use in their lifetime.

As shown in Table 4, prevalence of reporting an adverse/negative health effect from cannabis use became less common with age. All age groups except 45-54-year-olds were significantly more likely to report this outcome compared to adults aged 55 and older, with youth aged 16–19 being approximately 4.5 times more likely. Unlike most other outcomes tested, reporting an adverse/negative effect from cannabis use did not vary by education level in the adjusted model. Note that one of the top five adverse effects selected by consumers in both 2023 and 2024 CCS was drowsiness/lethargy [1, 38]. Given that this may be an expected effect of cannabis use, sensitivity analyses were conducted to examine adverse effects excluding those who selected drowsiness/lethargy only. The pattern of results was the same as that shown in Table 4, except that total outcome prevalence was slightly lower (26.3% instead of 30.1%; data not shown).

**Table 4:** Experienced an adverse or negative health effect from cannabis use ^a^ in the past 12 months, among past 12-month cannabis consumers, 2023–2024 Canadian Cannabis Survey (*n* = 6,879)

Covariate	Prevalence (%) (95% CI)	Adjusted Odds Ratio, (95% CI)	*p*-value
Overall	30.1 (28.9–31.3)	F(23, 6182) = 22.41	**< 0.0001**
**Age group (years)**		F(5,6199) = 41.09	**< 0.0001**
16–19	53.1 (49.0-57.2)	4.45 (3.37–5.89)	**< 0.0001**
20–24	50.0 (47.1–52.9)	3.76 (3.04–4.65)	**< 0.0001**
25–34	36.6 (33.5–39.8)	2.33 (1.88–2.89)	**< 0.0001**
35–44	25.7 (23.1–28.4)	1.66 (1.34–2.06)	**< 0.0001**
45–54	20.7 (18.1–23.6)	1.31 (1.04–1.66)	0.021
55 and older	16.3 (14.4–18.4)	--ref--	--ref--
**Sex**		F(1, 6203) = 1.79	0.182
Female	30.5^↑^ (28.8–32.3)	--ref--	--ref--
Male	29.7 (28.0-31.4)	1.34 (0.94–1.26)	0.182
**Gender modality**		F(1, 6203) = 2.43	0.119
Cisgender	29.1 (27.9–30.3)	--ref--	--ref--
Gender diverse	49.0 (43.3–54.8)	1.28 (0.94–1.74)	0.119
**Sexual orientation**		F(3,6201) = 4.73	0.003
Heterosexual (straight)	26.1 (24.8–27.4)	--ref--	--ref--
Homosexual (lesbian or gay)	43.0 (36.6–49.6)	1.46 (1.07–1.99)	0.018
Bisexual/other sexual identity	49.4 (45.8–53.0)	1.40 (1.14–1.71)	**0.001**
Unstated	30.0 (23.5–37.5)	1.04 (0.65–1.65)	0.874
**Ethnic group**		F(2, 6202) = 1.27	0.280
White (exclusive category)	29.1 (27.7–30.4)	--ref--	--ref--
Indigenous	30.9 (25.8–36.5)	0.81 (0.60–1.09)	0.165
Other ethnic group/unstated	34.2 (31.3–37.3)	1.07 (0.88–1.29)	0.499
**Highest education level**		F(2, 6202) = 0.56	0.570
High school or less	36.5^↓^ (34.3–38.7)	0.91 (0.76–1.08)	0.290
Trades/college or non-university diploma or certificate	26.4 (24.3–28.6)	0.97 (0.82–1.14)	0.679
At least some university	28.1 (26.2–30.1)	--ref--	--ref--
**Household income**		F(2, 6202) = 6.01	0.003
Less than $50,000	35.9 (33.1–38.7)	1.34 (1.12–1.60)	**0.001**
$50,000-$99,999	31.2 (29.0-33.6)	1.22 (1.05–1.42)	
$100,000 or more	26.3 (24.5–28.1)	--ref--	--ref--
**Mental health status**		F(1, 6203) = 38.09	**< 0.0001**
Fair or Poor	43.8 (41.3–46.2)	1.59 (1.37–1.85)	**< 0.0001**
Other response	24.8 (23.4–26.2)	--ref--	--ref--
**Community size**		F(2, 6202) = 5.51	0.004
Rural/small community (< 30,000)	26.2 (24.1–28.3)	--ref--	--ref--
Medium community (30,000–99,999)	29.9 (27.2–32.6)	1.13 (0.94–1.37)	0.203
Large community (100,000 or more)	32.5^↓^ (30.8–34.3)	1.30 (1.11–1.52)	**0.001**
**Immigration status**		F(1, 6203) = 3.99	0.046
Born outside of Canada	27.9 (24.7–31.3	--ref--	--ref--
Born in Canada	30.4 (29.2–31.8)	1.24 (1.00-1.54)	0.046
**Provincial/territorial cannabis retail model**		F(2, 6202) = 0.99	0.373
Hybrid (public & hybrid sales)	30.3 (28.7–32.0)	1.12 (0.95–1.32)	0.173
Private sales	30.2 (27.8–32.7)	1.06 (0.87–1.28)	0.562
Public (government-run) sales	29.4 (26.9–31.9)	--ref--	--ref--

Significant differences at *p* < 0.003 are bolded

↑Indicates value should be rounded up if rounded to a whole number

↓Indicates value should be rounded down if rounded to a whole number

Ref=reference group

^a^ Includes any of the following: nausea/vomiting; heart or blood pressure problems; feeling faint/passing out/loss of consciousness; anxiety/panic attack/rapid heartbeat; hallucinations/psychosis/flashbacks; dissociation or depersonalization; slowed breathing/lung problems; allergic reaction; confusion/disorientation; unusual behaviour (e.g., agitation, slurred speech); chest pain/discomfort; loss of coordination/unsteadiness/vertigo; headache; diarrhea; seizure; drowsiness/lethargy; muscle weakness; other negative effect

Outcomes that may suggest ‘problematic’ patterns of cannabis use included reporting that cannabis had a harmful impact on one’s life, scoring as having ‘impaired control’ over one’s cannabis use on the SDS, and needing/receiving professional help for cannabis use. The odds of any of these outcomes were all significantly higher among younger age groups compared to adults 55 and older, males, and those with poor/fair mental health. Having ‘impaired control’ was also more common among those with lower incomes and those living in larger communities (Additional File 2).

### Threats to personal or public safety

Engaging in outcomes considered threats to personal or public safety were reported by approximately 4 in 10 consumers (41.5%) overall. Often/always combining cannabis with at least one other drug (including tobacco and alcohol, among other drugs) in the past year was reported by 3 in 10 consumers (30.0%), and driving after cannabis use was reported by 16.8% of consumers. Relatively few consumers with a ‘hazardous’ occupation reported cannabis use at work or within 2 hours of work (5.4%) and only 3.3% of consumers reported an illicit source (illegal store, illegal website or dealer) to be their usual cannabis purchase source (Additional File 2).

Prevalence of combining cannabis with at least one other drug followed the typical pattern of most results mentioned above: it declined with age, and was more common in males, those with lower education levels, and those with poor mental health. It was also significantly more common among those living in PTs with public sales compared to private or hybrid sales. Odds of usually purchasing cannabis from an illicit source were higher among youth 16–19 (vs. 20-24-year-olds), males, those with fair/poor mental health, and those living in PTs with exclusively public sales versus hybrid/private sales (Additional File 2).

As shown in Table 5, driving after cannabis use had a slightly different profile. In addition to being more than 1.5 times more common among males versus females and those with lower education levels versus university education, consumers with the highest likelihood to drive after cannabis use tended to be 35–44 years of age (vs. 55 and older), be born in Canada, live in PTs with private retail sales and live in medium-sized (vs. large) communities. Adjusted odds ratios also suggested that consumers living in small/rural communities were somewhat more likely to drive after cannabis use, but this contrast did not reach statistical significance (*p* = 0.016).

**Table 5:** Prevalence of driving within 2 h of smoking/vaping cannabis or 4 h of ingesting cannabis edibles in the past 12 months, 2023–2024 Canadian Cannabis Survey (*n* = 7,033)

Covariate	Prevalence (%) (95% CI)	Adjusted Odds Ratio, (95% CI)	*p*-value
Overall	16.8 (15.9–17.8)	F(21, 6343) = 8.09	**< 0.0001**
**Age group (years)**		F(5, 6339) = 2.58	0.024
16–19	15.7 (13.0-18.9)	0.99 (0.71–1.37)	0.934
20–24	16.5↑ (14.6–18.8)	1.16 (0.91–1.48)	0.238
25–34	17.0 (14.7–19.5)	1.29 (1.01–1.65)	0.038
35–44	20.6 (18.3–23.0)	1.42 (1.14–1.78)	**0.002**
45–54	16.4 (14.1–19.0)	1.09 (0.86–1.40)	0.460
55 and older	14.6 (12.9–16.6)	--ref--	--ref--
**Sex**		F(1, 6343) = 53.53	**< 0.0001**
Female	12.4 (11.2–13.7)	--ref--	--ref--
Male	21.0 (19.5–22.5)	1.80 (1.54–2.10)	**< 0.0001**
**Gender modality**		F(1, 6343) = 0.98	0.321
Cisgender	17.0 (16.0–18.0)	--ref--	--ref--
Gender diverse	13.3 (9.8–17.9)	0.80 (0.52–1.24)	0.321
**Sexual orientation**		F(2,6342) = 0.87	0.420
Heterosexual (straight)	17.6 (16.6–18.8)	--ref--	--ref--
Homosexual/Unstated	15.6 (12.3–19.6)	0.95 (0.69–1.32)	0.762
Bisexual or other sexual identity	12.9 (10.7–15.4)	0.83 (0.63–1.09)	0.188
**Ethnic group**		F(2, 6342) = 1.15	0.317
White (exclusive category)	17.1 (16.0-18.3)	--ref--	--ref--
Indigenous	17.1 (13.2–21.9)	0.85 (0.59–1.23)	0.385
Other ethnic group/unstated	15.6 (13.5–18.0)	1.14 (0.92–1.42)	0.242
**Highest education level**		F(2, 6342) = 13.18	**< 0.0001**
High school or less	19.2 (17.5–21.1)	1.61 (1.30–1.98)	**< 0.0001**
Trades/college or non-university diploma or certificate	20.0 (18.1–22.0)	1.56 (1.29–1.89)	**< 0.0001**
At least some university	12.7 (11.4–14.2)	--ref--	--ref--
**Household income**		F(2, 6342) = 2.88	0.056
Less than $50,000	15.9 (14.0-18.2)	1.24 (1.00-1.53)	0.045
$50,000-$99,999	19.2 (17.4–21.2)	1.04 (0.84–1.28)	0.708
$100,000 or more	16.4 (15.0-17.9)	--ref--	--ref--
**Mental health status**		F(1, 6343) = 0.55	0.459
Fair or Poor	17.9 (16.1–19.8)	1.07 (0.90–1.27)	0.459
Other response	16.4 (15.3–17.6)	--ref--	--ref--
**Community size**		F(2,6342) = 6.92	**0.001**
Rural/small community (< 30,000)	18.9 (17.1–20.8)	1.24 (1.04–1.48)	0.016
Medium community (30,000–99,999)	19.7 (17.5–22.2)	1.43 (1.18–1.74)	**< 0.0001**
Large community (100,000 or more)	14.7 (13.4–16.0)	--ref--	--ref--
**Immigration status**		F(1, 6343) = 17.44	**< 0.0001**
Born outside of Canada	10.5↑ (8.4–13.0)	--ref--	--ref--
Born in Canada	17.8 (16.8–18.9)	1.84 (1.38–2.46)	**< 0.0001**
**Provincial/territorial cannabis retail model**		F(2, 6342) = 7.12	**0.0008**
Hybrid (public & hybrid sales)	15.8 (14.6–17.2)	1.06 (0.87–1.27)	0.568
Private sales	21.0 (18.9–23.2)	1.43 (1.15–1.77)	**0.001**
Public (government-run) sales	15.8 (13.9–17.9)	--ref--	--ref--

Significant differences at *p* < 0.003 are bolded

↑Indicates value should be rounded up if rounded to a whole number

↓Indicates value should be rounded down if rounded to a whole number

Ref=reference group

## Discussion

The current study was unique in its ability to identify subgroups who were likely to engage in higher risk cannabis use behaviours and/or report negative outcomes associated with their cannabis consumption, examining differences across a number of demographic and socioeconomic groups. The present analysis suggests that over three quarters of cannabis consumers endorsed at least one higher risk outcome, the most common being at least weekly cannabis use and the least common being ‘usually’ purchasing cannabis from an illicit source.

Consistent with subgroup differences in past-year cannabis use from CCS and CSUS, several outcomes assessed were more common among consumers who were younger; male; had a high school (or lower) education or a trades/non-university diploma as opposed to a university education; had lower income levels; and reported poorer mental health [10, 44]. For example, youth were substantially more likely to use inhalable cannabis extracts, report usually selecting inhalable/ingestible extracts and edibles/beverages with high THC levels, purchase cannabis illegally, report an adverse effect from cannabis use, report that cannabis had a negative impact on their life, score as having ‘impaired control’ on the SDS, regularly combine cannabis with other drugs, and purchase from illicit sources compared to older consumers. This is particularly concerning given that youth are a highly vulnerable population, and cannabis can interfere with brain development until approximately age 25 [45]. Moreover, protecting youth is a key objective of the *Cannabis Act* [5]. On the other hand, frequent cannabis use was *least* likely among youth aged 16–19 compared to older age groups. This may be partially due to the fact that this age group includes some individuals below the MLA for purchasing cannabis in their province/territory. Indeed, almost half of youth in this sample report accessing cannabis from social sources [1], which might make cannabis slightly more difficult to access and use frequently. In fact, prevalence rates for frequent use were highest and second-highest among 35-44- and 20-24-year-olds, respectively. Taken together, findings could be used to identify specific population subgroups who may benefit from more targeted public education or harm reduction initiatives, addressing certain higher risk behaviours prevalent among different subgroups. For instance, messaging and intervention programs could be directed toward youth-based settings (e.g., high schools, community centres); social media channels targeting not only youth but adults in their 20s to 40s; trades schools or companies primarily employing individuals in the skilled trades; socioeconomically deprived areas [46]; and mental health professionals and addictions centres, for dissemination to their patients.

Driving after cannabis use, which is an oft-cited safety concern associated with legalization, had a slightly different profile. Like other outcomes, and consistent with CSUS 2023 [10], it was more likely among males and those with lower education levels. However, it was also more likely among those born in Canada versus those who had immigrated, and was less likely among those living in large cities (likely associated with greater availability of public transportation and ride booking services in metropolitan areas). Public education initiatives aiming to prevent driving ‘high’ have typically targeted youth [47, 48]. In the current study, despite not reaching statistical significance (*p* = 0.027), driving after cannabis use was approximately 5 percentage points higher among adults aged 35–44 compared to youth aged 16–19. Similarly, Public Safety Canada found that from 2015 to 2022, adults 35 and older had the largest increase in drug-impaired driving incidents in Canada. In contrast, the number of incidents among younger people aged 24 and under decreased [49]. Given that the population of adults (and therefore adult drivers) in Canada substantially outpopulates the population of youth [50], the higher likelihood of adults to drive after cannabis use is concerning. Educating youth on the importance of driving sober remains an important harm reduction measure. However, these data suggest that future campaigns on cannabis-impaired driving could additionally focus on middle-aged adults.

Demographic and regional populations who are more likely to purchase cannabis from illicit sources may be of interest to policy makers and law enforcement. Consistent with the 2023 CSUS, the CCS reports that regularly purchasing cannabis from illicit sources was significantly higher among consumers reporting poor/fair mental health as well as among youth aged 16–19, many of whom are below the MLA to purchase cannabis, as mentioned earlier [10]. Illicit purchasing was also more common among those living in PTs with exclusively public sales compared to private/hybrid sales.

Lastly, a notable finding of this comprehensive analysis was that (with the exception of sex and age group) differences in outcome rates by individual-level characteristics often became non-significant after adjustment for other factors. This was especially true for gender modality and sexual orientation, where differences of 10–20 percentage points were non-significant in adjusted models. Existing literature points to higher rates of cannabis use and CUD among both sexual and gender minorities [51, 52]. However, experts have noted that protective and intersecting factors play important roles in rates of cannabis use, and that sexual orientation and gender identity should not be considered in isolation [52]. This highlights the importance of including these variables in models with other micro- and macro-level factors. Race/ethnicity is another individual-level factor that often became non-significant in adjusted models. Previous research shows higher rates of cannabis use in Indigenous compared to non-Indigenous peoples, with rates varying between First Nations, Metis and Inuit populations [53, 54]. Published CCS data show that individuals identifying as Indigenous, Latino and White had the highest rates of past 12-month cannabis use [13]. In the present analysis, individuals identifying as either Indigenous or White had the highest rates of several higher risk cannabis outcomes, but differences were non-significant. Small sample sizes precluded subgroup analysis of individual ethnicities, leading to a heterogenous ‘other/unstated’ ethnicity category as well as the inability to analyze First Nations, Metis and Inuit groups separately. These analytical constraints may have obscured real-world differences in outcomes. However, it is also possible that socio-economic standing (education and income) or factors related to the respondent’s location of residence (community size, cannabis retail sales model) played a stronger role in cannabis-use outcomes than gender modality, sexual orientation or race/ethnicity. Future studies with larger samples of people identifying as Black, Indigenous, and People of Colour should examine interactions between these intersecting factors.

### Limitations

First, this study was subject to usual limitations of self-report data, including social desirability and recall biases, although some research has shown that substance use behaviours reported in web surveys are not associated with social desirability scores [55]. Second, interactions between variables were not tested and could be an area for future research. Third, individuals may have different personality traits or motivators for using cannabis or engaging in higher risk behaviours. As these were not queried in the CCS, they could not be considered. Fourth, to bolster sample size, respondents who used cannabis for medical and/or non-medical purposes were included. This may have obscured differences between consumers who use cannabis primarily for medicinal versus recreational purposes.^8^ Examining these differences represents a direction for future research. Fifth, the question on number of hours ‘high’/stoned per usage day was only asked to respondents who used cannabis in the past 30 days, and questions on usual potency of cannabis products were only asked to consumers of particular product types (e.g., edibles/beverages, extracts). To avoid zero-inflation (i.e., significantly more zero-value observations than expected if respondents who were not shown these questions were coded as ‘no’), we opted not to present prevalence rates among all past 12-month consumers for these behaviours. Estimates provided for these variables therefore represent proportions of the smaller sample who contributed to the denominator (see Table 1). Sixth, because the pooled data present period averages, any differences between 2023 and 2024 are minimized (including those of four higher risk outcomes that differed significantly by survey cycle in Table 1: using cannabis during/before work or school; using inhalable extracts; using extracts with > 30% THC, and driving after cannabis use). The authors felt comfortable with this limitation given that the paper focused less on estimating population prevalence and more on identifying characteristics that increase a consumer’s likelihood to engage in higher risk outcomes. Lastly, due to the large number of tests conducted in this study, a conservative p-value of < 0.003 was used. This led to a finding of non-significance for several covariates with p-values of < 0.01 and < 0.05. Some of these subgroup differences nevertheless may be meaningful in the real world, and readers interested in specific population subgroups are urged to examine the prevalence estimates and confidence intervals reported in text and Supplementary Information.

## Conclusion

Study results may be used to tailor education initiatives and harm reduction programming to subgroups of cannabis consumers in Canada who are more likely to engage in higher risk cannabis behaviours or report negative outcomes associated with their cannabis use. The CCS 2023–2024 did not include a direct assessment of CUD. However, individuals identified as engaging in heavy patterns of cannabis use, those scoring above the threshold for psychological dependence on the SDS, and those reporting the need for professional help could meet the profile for individual or group treatment to reduce cannabis dependence. Psychotherapeutic treatments to reduce cannabis use – including motivational enhancement therapy, cognitive behavioral therapy, contingency management and multidimensional family therapy – have shown mixed results, as reviewed elsewhere [19]. For youth, substance use prevention programs such as life skills training may represent avenues for treatment and prevention at the school or individual levels. Life skills training typically aims to improve stress management, social skills and the ability to combat peer pressure. Life skills programs have shown promise in reducing the frequency of cannabis and other substance use among youth, particularly when conducted via mobile phone [56, 57]. Finally, at the environmental level, public education campaigns could focus further on specific subgroups identified in this study. For example, health associations or other entities could develop tailored advertising on social media around cannabis-impaired driving that is geared toward adults in their 30–40s and those living in rural areas.

## Supplementary Information

Below is the link to the electronic supplementary material.

Supplementary Material 1

Supplementary Material 2

## Data Availability

The datasets supporting the conclusions of this article are available in the Open Government repository; 2023 Canadian Cannabis Survey: https://open.canada.ca/data/en/dataset/6c240c79-c857-4fd4-bbd6-b1fd7f04fdbc; 2024 Canadian Cannabis Survey: https://open.canada.ca/data/en/dataset/2abe0796-c3e6-443b-a5c5-74e770e9fbe0.

## Footnotes

1 In Canada, cannabis product packages must prominently display one of a series of rotating health warning messages. These relate to use during pregnancy and breastfeeding, driving after cannabis use, and effects on physical and mental health. See: https://www.canada.ca/en/health-canada/services/drugs-medication/cannabis/laws-regulations/regulations-support-cannabis-act/health-warning-messages.html.

2 The term ‘weakly’ psychoactive is used because CBD-predominant products may contain small amounts of THC.

3 Note that respondents were asked to enter THC in mg per piece of edible or per drink, which may not reflect total THC per package.

4 These particular age groups correspond to the age groups used in the Canadian Cannabis Survey sample weights, which were based on region, sex and age group.

5 Gender modality was derived. Cis gender indicates reported gender identity corresponded to sex at birth; gender diverse indicates it did not (woman/girl + male sex; man/boy + female sex; or other gender identity + either sex). Gender modality was used rather than self-reported gender identity because the prevalence of ‘other gender identity’ was too low for subgroup analysis.

6 Other responses included Good/Very good/Excellent/Prefer not to say. The latter was grouped with positive mental health responses due to small cell sizes and to test prevalence of poor/fair mental health vs. all other responses. Sensitivity analyses indicated that dropping ‘prefer not to say’ had no meaningful impact on model results.

7 At the time of writing, cannabis sales models were as follows: Hybrid (private & public sales): British Columbia, Ontario, New Brunswick, Newfoundland and Labrador, Yukon territory; Exclusively private: Alberta, Manitoba, Northwest Territories, Nunavut, Saskatchewan; Exclusively public: Nova Scotia, Prince Edward Island, Quebec.

8 This distinction was made for models of frequent cannabis use, which excluded consumers of cannabis for exclusively medical purposes.

## Abbreviations

CBD: cannabidiol
CCS: Canadian Cannabis Survey
CSUS: Canadian Substance Use Survey
CUD: Cannabis Use Disorder
MLA: minimum legal age
PT: province/territory
SGBA Plus: Sex and Gender-Based Analysis Plus
SDS: Severity of Dependence Scale
THC: tetrahydrocannabinol
