# Supplementary material for: Higher risk cannabis use indicators among people living in Canada: a cross-sectional study examining the association with socio-demographic and socio-economic factors
Source: Subst Abuse Treat Prev Policy. 2026 Apr 6;21:30. doi: 10.1186/s13011-026-00722-9 (PMC13126716; doi:10.1186/s13011-026-00722-9)
Supplement: Supplementary file 1 — Supplementary Material 1 [file 13011_2026_722_MOESM1_ESM.docx]

**Sample characteristics, past 12-month consumers in the Canadian Cannabis Survey 2023-2024 (n=7,238)**

| **Variable** | **Frequency (n)** | **Prevalence (%), 95% CI** |
| --- | --- | --- |
| **Age group (years)** |  |  |
| 16-19 | 639 | 7.4 (6.8-8.0) |
| 20-24 | 1,311 | 11.9 (11.2-12.6) |
| 25-34 | 1,113 | 22.9 (21.6-21.1) |
| 35-44 | 1,298 | 18.4 (17.5-19.4) |
| 45-54 | 1,083 | 13.0 (12.2-13.8) |
| 55 and older | 1,794 | 26.4 (25.2-27.5) |
| **Sex** |  |  |
| Female | 3,518 | 48.4 (47.1-49.7) |
| Male | 3,720 | 51.6 (50.3-52.9) |
| **Gender modality** |  |  |
| Cisgender (reported gender identity matches sex at birth) | 6,857 | 94.9 (94.4-95.5) |
| Transgender/other gender identity | 381 | 5.1 (4.5-5.6) |
| **Sexual orientation** |  |  |
| Heterosexual (straight) | 5,732 | 79.6 (78.5-80.6) |
| Homosexual (lesbian or gay) | 288 | 4.0 (3.5-4.5) |
| Bisexual | 809 | 10.9 (10.1-11.7) |
| Other sexual identity | 202 | 2.8 (2.4-3.2) |
| Unstated | 207 | 2.8 (2.4-3.3) |
| **Ethnic group** |  |  |
| White (exclusive category) | 5,636 | 76.9 (75.8-78.0) |
| Indigenous | 365 | 4.5 (4.0-5.1) |
| Other response (other/multiple ethnicities, unstated) | 1,237 | 18.6 (17.5-19.6) |
| **Highest education level** |  |  |
| High school or less | 2,291 | 29.0 (27.8-30.1) |
| Trades/college or non-university diploma or certificate | 2,145 | 29.9 (28.8-31.2) |
| At least some university | 2,746 | 41.1 (39.8-42.4) |
| **Household income** |  |  |
| Less than $50,000 | 1,523 | 22.4 (21.3-23.5) |
| $50,000-$99,999 | 2,130 | 32.6 (31.3-33.9) |
| $100,000 or more | 1,960 | 45.1 (43.7-46.4) |
| **Mental health status** |  |  |
| Fair or Poor | 2,109 | 72.0 (70.8-73.1) |
| Other response (excellent, very good, good, unstated) | 5,129 | 28.0 (26.9-29.2) |
| **Community size** |  |  |
| Rural/small community (<30,000) | 2,379 | 29.2 (28.0-30.4) |
| Medium community (30,000-99,999) | 1,447 | 18.9 (17.9-19.9) |
| Large community (100,000 or more) | 3,342 | 52.0 (50.7-53.3) |
| **Immigration status** |  |  |
| Born outside of Canada | 892 | 13.8 (12.9-14.7) |
| Born in Canada | 6,336 | 86.2 (85.3-87.1) |
| **Provincial/territorial cannabis retail model** |  |  |
| Hybrid (public & hybrid sales) | 3,924 | 61.9 (60.7-63.1) |
| Private sales | 1,617 | 19.0 (18.1-19.9) |
| Public (government-run) sales | 1,697 | 19.1 (18.2-20.1) |
