# Supplementary material for: Higher risk cannabis use indicators among people living in Canada: a cross-sectional study examining the association with socio-demographic and socio-economic factors
Source: Subst Abuse Treat Prev Policy. 2026 Apr 6;21:30. doi: 10.1186/s13011-026-00722-9 (PMC13126716; doi:10.1186/s13011-026-00722-9)
Supplement: Supplementary file 2 — Supplementary Material 2 [file 13011_2026_722_MOESM2_ESM.docx]

**Prevalence estimates and regression results for remaining outcomes, past 12-month consumers in the Canadian Cannabis Survey 2023-2024**

Additional Table 1. Prevalence of using cannabis daily/almost daily, among those who consumed cannabis for non-medical purposes in the past 12 months, 2023-2024 Canadian Cannabis Survey (n=6,198)

| **Covariate** | **Prevalence (%) (95% CI)** | **Adjusted Odds Ratio, (95% CI)** | **p-value** |
| --- | --- | --- | --- |
| **Overall** | 23.4 (22.2-24.6) | F(22, 6589)=9.35 | **<0.0001** |
| **Age group (years)** |  | F(5, 6606)=5.07 | **<0.0001** |
| 16-19 | 20.8 (17.7-24.4) | --ref-- | --ref-- |
| 20-24 | 25.0 (22.6-27.6) | 1.56 (1.17-2.08) | 0.003 |
| 25-34 | 23.8 (21.1-26.7) | 1.90 (1.39-2.61) | **<0.0001** |
| 35-44 | 25.9 (23.2-28.7) | 2.19 (1.60-3.00) | **<0.0001** |
| 45-54 | 21.5^↓^ (18.7-24.6) | 1.78 (1.28-2.47) | **0.001** |
| 55 and older | 21.6 (19.2-24.3) | 1.76 (1.28-2.42) | **0.001** |
| **Sex** |  | F(1, 6610)=17.09 | **<0.0001** |
| Female | 20.8 (19.2-22.5) | --ref-- | --ref-- |
| Male | 25.6 (23.9-27.3) | 1.38 (1.19-1.61) | **<0.0001** |
| **Gender modality** |  | F(1, 6610)=1.91 | 0.167 |
| Cisgender | 23.3 (22.1-24.6) | --ref-- | --ref-- |
| Gender diverse | 23.9 (19.0-29.6) | 0.77 (0.53-1.12) | 0.167 |
| **Sexual orientation** |  | F(3, 6608)=0.41 | 0.748 |
| Heterosexual (straight) | 22.8 (21.5-24.1) | --ref-- | --ref-- |
| Homosexual (gay or lesbian) | 26.2 (20.6-32.8) | 1.17 (0.82-1.68) | 0.381 |
| Bisexual or other sexual identity | 25.1 (22.1-28.3) | 1.07 (0.85-1.35) | 0.553 |
| Unstated | 27.2 (20.3-35.4) | 1.17 (0.72-1.91) | 0.532 |
| **Ethnic group** |  | F(2, 6609)=0.96 | 0.383 |
| White (exclusive category) | 23.4 (22.0-24.7) | --ref-- | --ref-- |
| Indigenous | 32.4 (26.7-38.6) | 1.23 (0.89-1.71) | 0.216 |
| Other ethnic group/unstated | 21.3 (18.7-24.1) | 0.94 (0.75-1.18) | 0.599 |
| **Highest education level** |  | F(2, 6609)=30.49 | **<0.0001** |
| High school or less | 30.7 (28.5-33.0) | 2.19 (1.79-2.68) | **<0.0001** |
| Trades/college or non-university diploma or certificate | 26.0 (23.8-28.4) | 1.65 (1.37-1.99) | **<0.0001** |
| At least some university | 16.1 (14.5-17.8) | --ref-- | --ref-- |
|  |  |  |  |
| **Household income** |  | F(2, 6609)=12.86 | **<0.0001** |
| Less than $50,000 | 27.6 (24.9-30.5) | 1.43 (1.17-1.76) | **<0.0001** |
| $50,000-$99,999 | 27.6 (25.3-29.9) | 1.54 (1.29-1.82) | **<0.0001** |
| $100,000 or more | 18.1 (16.6-19.9) | --ref-- | --ref-- |
| **Mental health status** |  | F(1, 6610)=23.09 | **<0.0001** |
| Fair or Poor | 30.4 (28.1-32.9) | 1.51 (1.28-1.78) | **<0.0001** |
| Other response | 20.6 (19.3-22.0) | --ref-- | --ref-- |
| **Community size** |  | F(2, 6609)=1.32 | 0.268 |
| Rural/small community (<30,000) | 23.5^↑^ (21.4-25.8) | --ref-- | --ref-- |
| Medium community (30,000-99,999) | 24.6 (22.0-27.4) | 1.16 (0.95-1.43) | 0.149 |
| Large community (100,000 or more) | 22.8 (21.2-24.5) | 1.28 (0.95-1.34) | 0.168 |
| **Immigration status** |  | F(1, 6610)=8.05 | 0.005 |
| Born outside of Canada | 16.4 (13.6-19.6) | --ref-- | --ref-- |
| Born in Canada | 24.4 (23.2-25.7) | 1.49 (1.13-1.96) | 0.005 |
|  |  |  |  |
| **Provincial/territorial cannabis retail model** |  | F(2, 6609)=3.15 | 0.043 |
| Hybrid (public & hybrid sales) | 23.5 (22.0-25.2) | 1.21 (1.00-1.46) | 0.046 |
| Private sales | 26.6 (24.2-29.2) | 1.31 (1.05-1.62) | 0.015 |
| Public (government-run) sales | 19.7 (17.5-22.1) | --ref-- | --ref-- |

Significant differences at p<0.003 are bolded.

↑Indicates value should be rounded up if rounded to a whole number.

↓Indicates value should be rounded down if rounded to a whole number.

Ref=reference group

Additional Table 2. Reported being ‘high/stoned’ for ≥5 hours per usage day in the past 30 days, among past 30-day cannabis consumers, 2023-2024 Canadian Cannabis Survey (n=4,678)

| **Covariate** | **Prevalence (%) (95% CI)** | **Adjusted Odds Ratio, (95% CI)** | **p-value** |
| --- | --- | --- | --- |
| **Overall** | 16.7 (15.5-17.9) | F(22,4199)=4.57 | **<0.0001** |
| **Age group (years)** |  | F(5,4216)=2.10 | 0.063 |
| 16-19 | 17.6 (14.2-21.7) | --ref-- | --ref-- |
| 20-24 | 20.8 (18.1-23.8) | 1.53 (1.03-2.27) | 0.034 |
| 25-34 | 19.6 (16.6-23.0) | 1.69 (1.11-2.58) | 0.014 |
| 35-44 | 14.3 (11.9-17.0) | 1.23 (0.80-1.89) | 0.353 |
| 45-54 | 16.7 (13.9-19.9) | 1.62 (1.05-2.48) | 0.029 |
| 55 and older | 13.6 (11.5-16.0) | 1.33 (0.87-2.04) | 0.182 |
| **Sex** |  | F(1, 4420)=31.81 | **<0.0001** |
| Female | 13.2 (11.6-14.9) | --ref-- | --ref-- |
| Male | 19.7 (18.0-21.5) | 1.83 (1.48-2.50) | **<0.0001** |
| **Gender modality** |  | F(1, 4420)=2.64 | 0.104 |
| Cisgender | 16.6 (15.4-17.8) | --ref-- | --ref-- |
| Gender diverse | 18.5↓ (13.7-24.4) | 0.68 (0.43-1.08) | 0.104 |
| **Sexual orientation** |  | F(3, 4218)=2.14 | 0.093 |
| Heterosexual (straight) | 15.7 (14.4-17.0) | --ref-- | --ref-- |
| Homosexual (lesbian or gay) | 17.1 (12.9-22.4) | 1.05 (0.64-1.73) | 0.853 |
| Bisexual/other sexual identity | 21.6 (18.3-25.3) | 1.43 (1.06-1.93) | 0.604 |
| Unstated |  | 1.47 (0.78-2.78) | 0.234 |
| **Ethnic group** |  | F(2, 4219)=1.99 | 0.137 |
| White (exclusive category) | 15.8 (14.5-17.1) | --ref-- | --ref-- |
| Indigenous | 19.7 (14.8-25.8) | 1.18 (0.80-1.75) | 0.403 |
| Other ethnic group/unstated | 19.7 (16.8-23.1) | 1.30 (0.99-1.71) | 0.061 |
| **Highest education level** |  | F(2, 4219)=2.75 | 0.064 |
| High school or less | 20.5^↓^ (18.4-22.8) | 1.36 (1.05-1.76) | 0.019 |
| Trades/college or non-university diploma or certificate | 15.9 (13.9-18.1) | 1.16 (0.90-1.48) | 0.248 |
| At least some university | 13.9 (12.1-16.0) | --ref-- | --ref-- |
|  |  |  |  |
| **Household income** |  | F(2, 4219)=8.92 | **0.0001** |
| Less than $50,000 | 21.3 (18.6-24.3) | 1.68 (1.31-2.16) | **<0.0001** |
| $50,000-$99,999 | 18.1 (15.9-20.6) | 1.39 (1.11-1.74) | 0.004 |
| $100,000 or more | 12.6 (11.0-14.3) | --ref-- | --ref-- |
| **Mental health status** |  | F(1, 4420)=15.73 | **0.0001** |
| Fair or Poor | 22.6 (20.3-25.2) | 1.52 (1.24-1.88) | **<0.0001** |
| Other response | 14.1 (12.8-15.5) | --ref-- | --ref-- |
| **Community size** |  | F(2, 4219)=0.02 | 0.981 |
| Rural/small community (<30,000) | 16.4 (14.4-18.7) | --ref-- | --ref-- |
| Medium community (30,000-99,999) | 16.9 (14.4-19.8) | 1.00 (0.77-1.31) | 0.980 |
| Large community (100,000 or more) | 16.8 (15.1-18.6) | 1.02 (0.82-1.28) | 0.858 |
| **Immigration status** |  | F(1, 4420)=0.77 | 0.380 |
| Born outside of Canada | 16.8 (15.6-18.1) | --ref-- | --ref-- |
| Born in Canada | 15.2 (11.9-19.2) | 1.17 (0.82-1.67) | 0.380 |
|  |  |  |  |
| **Provincial/territorial cannabis retail model** |  | F(2, 4219)=0.77 | 0.464 |
| Hybrid (public & hybrid sales) | 17.0 (15.4-18.7) | 1.16 (0.90-1.48) | 0.237 |
| Private sales | 17.6 (15.3-20.1) | 1.16 (0.88-1.54) | 0.296 |
| Public (government-run) sales | 14.5^↓^ (12.2-17.1) | --ref-- | --ref-- |

Significant differences at p<0.003 are bolded.

↑Indicates value should be rounded up if rounded to a whole number.

↓Indicates value should be rounded down if rounded to a whole number.

Ref=reference group

Additional Table 3. Regularly used cannabis at work or school ^a^ in the past 12 months, among past 12-month cannabis consumers, 2023-2024 Canadian Cannabis Survey (n=6,267)

| **Covariate** | **Prevalence (%) (95% CI)** | **Adjusted Odds Ratio, (95% CI)** | **p-value** |
| --- | --- | --- | --- |
| **Overall** | 6.6 (6.8-8.8) | F(21, 6471)=11.79 | **<0.0001** |
| **Age group (years)** |  | F(55, 6487)=7.15 | **<0.0001** |
| 16-19 | 16.0 (13.2-19.2) | --ref-- | --ref-- |
| 20-24 | 10.7 (9.1-12.6) | 0.73 (0.51-1.04) | 0.079 |
| 25-34 | 7.0 (5.6-8.8) | 0.64 (0.42-0.96) | 0.033 |
| 35-44 | 6.7 (5.3-8.3) | 0.58 (0.39-0.88) | 0.009 |
| 45-54 | 5.6 (4.2-7.4) | 0.57 (0.36-0.90) | 0.017 |
| 55 and older | 2.2^E^ (1.5-3.1) | 0.21 (0.13-0.36) | **<0.0001** |
| **Sex** |  | F(1, 6491)=29.13 | **<0.0001** |
| Female | 5.0 (4.3-5.9) | --ref-- | --ref-- |
| Male | 8.1 (7.2-9.2) | 1.98 (1.54-2.53) | **<0.0001** |
| **Gender modality** |  | F(1, 6491)=2.00 | 0.157 |
| Cisgender | 6.3 (5.6-6.9) | --ref-- | --ref-- |
| Gender diverse | 13.6 (10.3-17.9) | 1.38 (0.88-2.17) | 0.157 |
| **Sexual orientation** |  | F(2, 6490)=0.61 | 0.544 |
| Heterosexual (straight) | 5.7 (5.1-6.4) | --ref-- | --ref-- |
| Homosexual/Unstated | 10.7 (8.0-14.2) | 1.27 (0.82-1.97) | 0.282 |
| Bisexual/other sexual identity | 9.9 (8.1-12.1) | 1.10 (0.77-1.57) | 0.604 |
| **Ethnic group** |  | F(2, 6490)=2.29 | 0.101 |
| White (exclusive category) | 6.0 (5.4-6.8) | --ref-- | --ref-- |
| Indigenous | 10.5^↓^ (7.6-14.4) | 1.24 (0.76-2.02) | 0.376 |
| Other ethnic group/unstated | 8.1 (6.6-9.9) | 1.38 (1.01-1.89) | 0.042 |
| **Highest education level** |  | F(2, 6490)=10.61 | **<0.0001** |
| High school or less | 11.0 (9.7-12.5) | 2.18 (1.55-3.07) | **<0.0001** |
| Trades/college or non-university diploma or certificate | 6.2 (5.2-7.5) | 1.77 (1.28-2.44) | **0.001** |
| At least some university | 3.6 (2.9-4.5) | --ref-- | --ref-- |
|  |  |  |  |
| **Household income** |  | F(2, 6490)=1.58 | 0.207 |
| Less than $50,000 | 8.1 (6.7-9.8) | 1.29 (0.96-1.74) | 0.096 |
| $50,000-$99,999 | 6.8 (5.7-8.0) | 1.21 (0.92-1.59) | 0.180 |
| $100,000 or more | 5.2 (4.4-6.2) | --ref-- | --ref-- |
| **Mental health status** |  | F(1, 6491)=21.33 | **<0.0001** |
| Fair or Poor | 11.6 (10.1-13.1) | 1.79 (1.40-2.30) | **<0.0001** |
| Other response | 4.7 (4.1-5.4) | --ref-- | --ref-- |
| **Community size** |  | F(2, 6490)=1.01 | 0.363 |
| Rural/small community (<30,000) | 6.2 (5.2-7.4) | --ref-- | --ref-- |
| Medium community (30,000-99,999) | 6.4 (5.1-7.9) | 1.05 (0.76-1.45) | 0.776 |
| Large community (100,000 or more) | 6.9 (6.0-7.8) | 1.21 (0.92-1.57) | 0.177 |
| **Immigration status** |  | F(1, 6491)=3.37 | 0.067 |
| Born outside of Canada | 4.6 (3.3-6.4) | --ref-- | --ref-- |
| Born in Canada | 6.9 (6.3-7.6) | 1.50 (0.97-2.31) | 0.067 |
|  |  |  |  |
| **Provincial/territorial cannabis retail model** |  | F(2, 6490)=0.78 | 0.460 |
| Hybrid (public & hybrid sales) | 6.6 (5.8-7.5) | 0.94 (0.71-1.25) | 0.692 |
| Private sales | 6.6 (5.5-8.0) | 0.81 (0.58-1.44) | 0.236 |
| Public (government-run) sales | 6.8 (5.6-8.3) | --ref-- | --ref-- |

Significant differences at p<0.003 are bolded.

↑Indicates value should be rounded up if rounded to a whole number.

↓Indicates value should be rounded down if rounded to a whole number.

Ref=reference group

^E^ Estimate should be interpreted with caution due to moderate sampling variability.

^a^ Sometimes, often or always used cannabis within 2h of work/school or during work/school in the past 12 months.

Additional Table 4. Typically selects THC-predominant products ^a^, among past 12-month cannabis consumers, 2023-2024 Canadian Cannabis Survey (n=7,208)

| **Covariate** | **Prevalence (%) (95% CI)** | **Adjusted Odds Ratio, (95% CI)** | **p-value** |
| --- | --- | --- | --- |
| **Overall** | 34.9 (33.7-36.1) | F(22, 6487)=9.13 | **<0.0001** |
| **Age group (years)** |  | F(5, 6504)=2.25 | 0.047 |
| 16-19 | 41.4 (37.5-45.5) | 1.29 (1.00-1.66) | 0.055 |
| 20-24 | 39.0 (36.2-41.8) | 1.31 (1.09-1.58) | 0.005 |
| 25-34 | 35.7 (32.7-38.8) | 1.19 (0.98-1.43) | 0.076 |
| 35-44 | 35.4 (32.6-38.3) | 1.10 (0.92-1.32) | 0.286 |
| 45-54 | 32.0 (29.1-35.2) | 1.00 (0.83-1.21) | 0.995 |
| 55 and older | 31.5^↓^ (29.2-33.9) | --ref-- | --ref-- |
| **Sex** |  | F(1, 6508)=89.87 | **<0.0001** |
| Female | 28.1 (26.5-29.8) | --ref-- | --ref-- |
| Male | 41.3 (39.5-43.0) | 1.80 (1.60-2.04) | **<0.0001** |
| **Gender modality** |  | F(1, 6508)=1.42 | 0.233 |
| Cisgender | 35.1 (33.8-36.4) | --ref-- | --ref-- |
| Gender diverse | 31.2 (26.2-36.7) | 0.83 (0.60-1.13) | 0.233 |
| **Sexual orientation** |  | F(3, 6506)=1.78 | 0.148 |
| Heterosexual (straight) | 35.5^↑^ (34.1-36.9) | --ref-- | --ref-- |
| Homosexual (lesbian or gay) | 32.9 (27.2-39.3) | 0.77 (0.57-1.05) | 0.097 |
| Bisexual/other sexual identity | 33.2 (30.0-36.6) | 0.87 (0.71-1.07) | 0.178 |
| Unstated | 28.4 (22.0-35.9) | 0.74 (0.48-1.13) | 0.167 |
| **Ethnic group** |  | F(2, 6507)=1.45 | 0.234 |
| White (exclusive category) | 34.8 (33.5-36.2) | --ref-- | --ref-- |
| Indigenous | 32.2 (27.0-37.9) | 0.82 (0.61-1.10) | 0.184 |
| Other ethnic group/unstated | 35.7 (32.8-38.8) | 1.09 (0.92-1.28) | 0.337 |
| **Highest education level** |  | F(2, 6507)=14.74 | **<0.0001** |
| High school or less | 41.2 (39.0-43.5) | 1.53 (1.30-1.80) | **<0.0001** |
| Trades/college or non-university diploma or certificate | 36.5 (34.2-38.8) | 1.34 (1.15-1.55) | **<0.0001** |
| At least some university | 29.2 (27.4-31.2) | --ref-- | --ref-- |
|  |  |  |  |
| **Household income** |  | F(2, 6507)=2.16 | 0.116 |
| Less than $50,000 | 38.4 (35.7-41.2) | 1.18 (1.01-1.39) | 0.040 |
| $50,000-$99,999 | 34.9 (32.7-37.2) | 1.04 (0.91-1.20) | 0.562 |
| $100,000 or more | 33.9 (32.1-35.9) | --ref-- | --ref-- |
| **Mental health status** |  | F(1, 6508)=4.23 | 0.040 |
| Fair or Poor | 38.1 (35.7-40.4) | 1.16 (1.01-1.33) | 0.040 |
| Other response | 33.6 (32.2-35.1) | --ref-- | --ref-- |
| **Community size** |  | F(2, 6507)=3.80 | 0.022 |
| Rural/small community (<30,000) | 34.0 (31.8-36.3) | --ref-- | --ref-- |
| Medium community (30,000-99,999) | 33.9 (31.2-36.7) | 1.04 (0.88-1.24) | 0.628 |
| Large community (100,000 or more) | 35.7 (34.0-37.5) | 1.20 (1.05-1.38) | 0.009 |
| **Immigration status** |  | F(1, 6508)=5.74 | 0.017 |
| Born outside of Canada | 31.5^↓^ (28.2-35.0) | --ref-- | --ref-- |
| Born in Canada | 35.4 (34.1-36.8) | 1.27 (1.04-1.54) | 0.017 |
|  |  |  |  |
| **Provincial/territorial cannabis retail model** |  | F(2, 6507)=1.52 | 0.219 |
| Hybrid (public & hybrid sales) | 33.9 (32.3-35.6) | 0.89 (0.77-1.03) | 0.109 |
| Private sales | 36.1 (33.6-38.6) | 0.96 (0.81-1.15) | 0.683 |
| Public (government-run) sales | 36.8 (34.2-39.5) | --ref-- | --ref-- |

↑Indicates value should be rounded up if rounded to a whole number.

↓Indicates value should be rounded down if rounded to a whole number.

Ref=reference group

^a^ Respondent selected ‘Higher THC, lower CBD’ or ‘THC only’ when asked which cannabis products they typically choose. Other response options included ‘CBD only’, ‘Higher CBD, lower THC’, ‘Equal levels of THC and CBD’, ‘A mix of the products above’, ‘Other’ and ‘Don’t know/not sure’.

Additional Table 5. Usually consumes cannabis with potency of >30% THC, ^a^ among past 12-month cannabis consumers of inhalable/ingestible extracts, 2023-2024 Canadian Cannabis Survey (n=1,405)

| **Covariate** | **Prevalence (%) (95% CI)** | **Adjusted Odds Ratio, (95% CI)** | **p-value** |
| --- | --- | --- | --- |
| **Overall** | 49.0 (46.1-52.0) | F(21, 1259)=6.74 | **<0.0001** |
| **Age group (years)** |  | F(5, 1275)=16.82 | **<0.0001** |
| 16-19 | 82.5^↑^ (75.3-88.0) | 9.16 (4.84-17.4) | **<0.0001** |
| 20-24 | 70.9 (65.3-76.0) | 6.29 (3.91-10.12) | **<0.0001** |
| 25-34 | 51.6 (45.0-58.1) | 2.94 (1.87-4.62) | **<0.0001** |
| 35-44 | 41.8 (35.3-48.7) | 1.79 (1.14-2.82) | 0.012 |
| 45-54 | 38.4 (31.0-46.4) | 1.59 (0.98-2.57) | 0.059 |
| 55 and older | 26.1 (20.5-32.4) | --ref-- | --ref-- |
| **Sex** |  | F(1, 1279)=9.41 | **0.002** |
| Female | 42.4 (37.8-47.2) | --ref-- | --ref-- |
| Male | 53.1 (49.3-56.8) | 1.59 (1.18-2.14) | **0.002** |
| **Gender modality** |  | F(1, 1279)=0.04 | 0.837 |
| Cisgender | 48.6 (45.5-51.6) | --ref-- | --ref-- |
| Gender diverse | 54.9 (43.5-65.9) | 1.07 (0.55-2.11) | 0.837 |
| **Sexual orientation** |  | F(2, 1278)=1.00 | 0.370 |
| Heterosexual (straight) | 47.5^↑^ (44.2-50.9) | --ref-- | --ref-- |
| Homosexual/Prefer not to say | 46.1 (35.6-56.9) | 0.72 (0.40-1.29) | 0.268 |
| Bisexual/other sexual identity | 57.2 (49.8-64.4) | 0.78 (0.49-1.24) | 0.286 |
| **Ethnic group** |  | F(2, 1278)=0.24 | 0.787 |
| White (exclusive category) | 48.2 (44.9-51.6) | --ref-- | --ref-- |
| Indigenous | 55.8 (44.4-66.6) | 1.20 (0.70-2.06) | 0.516 |
| Other ethnic group/unstated | 50.1 (42.6-57.6) | 0.96 (0.62-1.48) | 0.844 |
| **Highest education level** |  | F(2, 1278)=2.64 | 0.072 |
| High school or less | 60.1 (55.4-64.6) | 1.53 (1.06-2.19) | 0.022 |
| Trades/college or non-university diploma or certificate | 45.3 (40.0-50.7) | 1.27 (0.89-1.80) | 0.189 |
| At least some university | 39.5↓ (34.2-45.0) | --ref-- | --ref-- |
|  |  |  |  |
| **Household income** |  | F(2, 1278)=0.84 | 0.430 |
| Less than $50,000 | 48.3 (42.3-54.3) | 0.79 (0.55-1.13) | 0.195 |
| $50,000-$99,999 | 47.1 (41.9-52.4) | 0.93 (0.67-1.28) | 0.640 |
| $100,000 or more | 49.0 (44.2-53.9) | --ref-- | --ref-- |
| **Mental health status** |  | F(1, 1279)=2.37 | 0.124 |
| Fair or Poor | 58.7 (53.7-63.6) | 1.28 (0.94-1.74) | 0.124 |
| Other response | 44.0 (40.4-47.6) | --ref-- | --ref-- |
| **Community size** |  | F(2, 1278)=0.28 | 0.757 |
| Rural/small community (<30,000) | 50.0 (44.7-55.4) | --ref-- | --ref-- |
| Medium community (30,000-99,999) | 47.6 (41.0-54.3) | 0.86 (0.58-1.29) | 0.472 |
| Large community (100,000 or more) | 48.8 (44.6-52.9) | 0.97 (0.70-1.34) | 0.853 |
| **Immigration status** |  | F(1, 1279)=1.34 | 0.248 |
| Born outside of Canada | 50.1 (47.0-53.2) | --ref-- | --ref-- |
| Born in Canada | 39.1 (30.0-49.1) | 1.36 (0.81-2.27) | 0.248 |
|  |  |  |  |
| **Provincial/territorial cannabis retail model** |  | F(2, 1278)=12.85 | **<0.0001** |
| Hybrid (public & hybrid sales) | 51.0 (46.9-55.1) | 2.29 (1.58-3.32) | **<0.0001** |
| Private sales | 56.5↓ (51.0-61.7) | 2.71 (1.81-4.06) | **<0.0001** |
| Public (government-run) sales | 32.7 (27.1-38.9) | --ref-- | --ref-- |

Significant differences at p<0.003 are bolded.

↑Indicates value should be rounded up if rounded to a whole number.

↓Indicates value should be rounded down if rounded to a whole number.

Ref=reference group

^a^ Among respondents who had consumed hash/kief, vape pens/cartridges, concentrates/extracts or oils for oral use in the past 12 months and entered a usual THC concentration for their products in %THC or mg/g THC. Excludes those who selected ‘Don’t know’ or who entered a THC concentration in mg (because product weight/volume was unknown).

Additional Table 6. Usually consumes edibles/beverages with >10 mg THC per unit, ^a^ among past 12-month cannabis consumers, 2023-2024 Canadian Cannabis Survey (n=2,097)

| **Covariate** | **Prevalence (%) (95% CI)** | **Adjusted Odds Ratio, (95% CI)** | **p-value** |
| --- | --- | --- | --- |
| **Overall** | 25.3 (23.2-27.4) | F(21, 1917)=7.62 | **<0.0001** |
| **Age group (years)** |  | F(5, 1933)=2.82 | 0.015 |
| 16-19 | 47.5^↑^ (39.7-55.5) | --ref-- | --ref-- |
| 20-24 | 27.7 (23.6-32.2) | 0.43 (0.28-0.69) | **<0.0001** |
| 25-34 | 22.9 (18.5-27.9) | 0.44 (0.26-0.75) | **0.002** |
| 35-44 | 21.6 (17.6-26.3) | 0.44 (0.26-0.73) | **0.002** |
| 45-54 | 22.7 (17.9-28.4) | 0.49 (0.28-0.83) | 0.009 |
| 55 and older | 24.6 (20.2-29.6) | 0.50 (0.30-0.84) | 0.008 |
| **Sex** |  | F(1, 1937)=28.56 | **<0.0001** |
| Female | 19.0 (16.5-21.8) | --ref-- | --ref-- |
| Male | 31.4 (28.3-34.6) | 2.04 (1.57-2.64) | **<0.0001** |
| **Gender modality** |  | F(1, 1937)=1.80 | 0.180 |
| Cisgender | 24.6 (22.5-26.8) | --ref-- | --ref-- |
| Gender diverse | 34.3 (26.1-43.5) | 1.44 (0.85-2.45) | 0.180 |
| **Sexual orientation** |  | F(2, 1936)=2.17 | 0.115 |
| Heterosexual (straight) | 25.6 (23.2-28.1) | --ref-- | --ref-- |
| Homosexual/ | 26.9 (19.6-35.6) | 0.84 (0.51-1.39) | 0.494 |
| Bisexual/other sexual identity | 23.4 (19.0-28.5) | 0.65 (0.43-0.98) | 0.038 |
| **Ethnic group** |  | F(2, 1936)=0.92 | 0.399 |
| White (exclusive category) | 24.3 (22.1-26.7) | --ref-- | --ref-- |
| Indigenous | 30.4 (21.7-40.8) | 1.30 (0.72-2.35) | 0.377 |
| Other ethnic group/unstated | 28.3 (23.1-34.2) | 1.22 (0.85-1.76) | 0.276 |
| **Highest education level** |  | F(2, 1936)=12.25 | **<0.0001** |
| High school or less | 36.3 (32.3-40.6) | 2.05 (1.44-2.92) | **<0.0001** |
| Trades/college or non-university diploma or certificate | 30.1 (26.1-34.5) | 2.11 (1.53-2.92) | **<0.0001** |
| At least some university | 15.2 (12.7-18.1) | --ref-- | --ref-- |
|  |  |  |  |
| **Household income** |  | F(2, 1936)=7.79 | **0.0004** |
| Less than $50,000 | 36.2 (30.9-41.9) | 1.95 (1.39-2.74) | **<0.0001** |
| $50,000-$99,999 | 27.1 (23.2-31.4) | 1.37 (1.02-1.83) | 0.036 |
| $100,000 or more | 20.2 (17.6-23.1) | --ref-- | --ref-- |
| **Mental health status** |  | F(1, 1937)=11.96 | **0.0006** |
| Fair or Poor | 32.9 (29.0-37.0) | 1.63 (1.23-2.14) | **0.0006** |
| Other response | 21.8 (19.5-24.3) | --ref-- | --ref-- |
| **Community size** |  | F(2, 1936)=0.72 | 0.488 |
| Rural/small community (<30,000) | 30.4 (26.5-34.7) | --ref-- | --ref-- |
| Medium community (30,000-99,999) | 26.6 (21.9-31.8) | 1.20 (0.89-1.61) | 0.235 |
| Large community (100,000 or more) | 22.1 (19.4-24.9) | 1.05 (0.76-1.46) | 0.763 |
| **Immigration status** |  | F(1, 1937)=0.57 | 0.451 |
| Born outside of Canada | 21.1 (15.8-27.8) | --ref-- | --ref-- |
| Born in Canada | 25.6 (23.5-27.9) | 1.18 (0.77-1.79) | 0.451 |
|  |  |  |  |
| **Provincial/territorial cannabis retail model** |  | F(2, 1936)=7.04 | **0.0009** |
| Hybrid (public & hybrid sales) | 26.1 (23.5-28.8) | 1.55 (1.13-2.11) | 0.006 |
| Private sales | 18.5^↓^ (15.1-22.4) | --ref-- | --ref-- |
| Public (government-run) sales | 33.5^↓^ (27.8-39.7) | 2.12 (1.42-3.17) | **<0.0001** |

Significant differences at p<0.003 are bolded.

↑Indicates value should be rounded up if rounded to a whole number.

↓Indicates value should be rounded down if rounded to a whole number.

Ref=reference group

^a^ Among respondents who had consumed cannabis edibles or beverages in the past 12 months and entered a usual THC concentration for their products in mg THC per unit. Excludes those who selected ‘Don’t know’.

Additional Table 7. Reported cannabis had a somewhat or very harmful impact on their life ^a^ in the past 12 months, among past 12-month cannabis consumers, 2023-2024 Canadian Cannabis Survey (n=7,180)

| **Covariate** | **Prevalence (%) (95% CI)** | **Adjusted Odds Ratio, (95% CI)** | **p-value** |
| --- | --- | --- | --- |
| **Overall** | 19.1 (18.1-20.1) | F(23, 6461)=14.95 | **<0.0001** |
| **Age group (years)** |  | F(5,6479)=31.21 | **<0.0001** |
| 16-19 | 34.7 (30.9-38.7) | 4.27 (3.14-5.82) | **<0.0001** |
| 20-24 | 33.2 (30.6-36.0) | 3.82 (3.00-4.88) | **<0.0001** |
| 25-34 | 22.4 (19.8-25.2) | 2.26 (1.78-2.91) | **<0.0001** |
| 35-44 | 17.4 (15.2-19.9) | 1.84 (1.44-2.36) | **<0.0001** |
| 45-54 | 12.4 (10.4-14.7) | 1.33 (1.01-1.76) | 0.041 |
| 55 and older | 9.7 (8.2-11.3) | --ref-- | --ref-- |
| **Sex** |  | F(1, 6483)=25.95 | **<0.0001** |
| Female | 16.8 (15.5-18.3) | --ref-- | --ref-- |
| Male | 21.2 (19.7-22.7) | 1.50 (1.28-1.75) | **<0.0001** |
| **Gender modality** |  | F(1, 6483)=0.57 | 0.449 |
| Cisgender | 18.9 (17.8-19.9) | --ref-- | --ref-- |
| Gender diverse | 22.9 (18.5-27.8) | 0.87 (0.61-1.24) | 0.449 |
| **Sexual orientation** |  | F(4, 6480)=0.13 | 0.970 |
| Heterosexual (straight) | 17.7 (16.7-18.9) | --ref-- | --ref-- |
| Homosexual (lesbian or gay) | 23.1 (18.0-29.0) | 0.95 (0.66-1.36) | 0.784 |
| Bisexual | 26.9 (23.6-30.5) | 1.02 (0.80-1.29) | 0.896 |
| Other sexual identity | 23.4 (17.6-30.4) | 0.88 (0.56-1.37) | 0.563 |
| Unstated ^E^ | 15.8 (11.1-22.0) | 0.92 (0.56-1.52) | 0.744 |
| **Ethnic group** |  | F(2, 6482)=1.22 | 0.294 |
| White (exclusive category) | 18.7 (14.6-23.6) | --ref-- | --ref-- |
| Indigenous | 18.1 (17.0-19.3) | 0.85 (0.60-1.21) | 0.376 |
| Other ethnic group/unstated | 23.2 (20.7-25.9) | 1.13 0.93-1.39) | 0.224 |
| **Highest education level** |  | F(2, 6482)=0.70 | 0.497 |
| High school or less | 23.6 (21.7-25.5) | 0.89 (0.73-1.09) | 0.255 |
| Trades/college or non-university diploma or certificate | 16.9 (15.2-18.8) | 0.92 (0.76-1.12) | 0.414 |
| At least some university | 17.5^↓^ (15.9-19.1) | --ref-- | --ref-- |
|  |  |  |  |
| **Household income** |  | F(2, 6482)=4.85 | 0.008 |
| Less than $50,000 | 22.7 (20.4-25.2) | 1.34 (1.10-1.63) | 0.003 |
| $50,000-$99,999 | 19.9 (18.0-21.9) | 1.22 (1.02-1.45) | 0.028 |
| $100,000 or more | 16.7 (15.3-18.3) | --ref-- | --ref-- |
| **Mental health status** |  | F(1, 6483)=48.66 | **<0.0001** |
|  |  |  |  |
| Fair or Poor | 28.4 (26.3-30.6) | 1.77 (1.51-2.09) | **<0.0001** |
| Other response | 15.4 (14.3-16.6) | --ref-- | --ref-- |
| **Community size** |  | F(2, 6482)=2.09 | 0.123 |
| Rural/small community (<30,000) | 17.1 (15.4-18.9) | --ref-- | --ref-- |
| Medium community (30,000-99,999) | 20.4 (18.1-22.8) | 1.22 (0.98-1.50) | 0.071 |
| Large community (100,000 or more) | 19.7 (18.3-21.2) | 1.17 (0.98-1.40) | 0.076 |
| **Immigration status** |  | F(1, 6483)=0.68 | 0.411 |
| Born outside of Canada | 19.1 (18.0-20.2) | --ref-- | --ref-- |
| Born in Canada | 19.0 (16.3-22.1) | 1.11 (0.87-1.41) | 0.411 |
|  |  |  |  |
| **Provincial/territorial cannabis retail model** |  | F(2, 6482)=0.85 | 0.426 |
| Hybrid (public & hybrid sales) | 19.4 (18.1-20.8) | 1.04 (0.86-1.25) | 0.688 |
| Private sales | 17.8 (15.9-19.9) | 0.92 (0.74-1.15) | 0.463 |
| Public (government-run) sales | 19.2 (17.2-21.5) | --ref-- | --ref-- |

Significant differences at p<0.003 are bolded.

↑Indicates value should be rounded up if rounded to a whole number.

↓Indicates value should be rounded down if rounded to a whole number.

Ref=reference group

^a^ Includes impact on any of the following: friendships or social life; physical health; mental health; home life or marriage; performance at work or school; and quality of life.

Additional 8. Prevalence of scoring as having ‘impaired control’ over one’s cannabis use on Severity of Dependence Scale, among those who have consumed cannabis in the past 12 months, 2023-2024 Canadian Cannabis Survey (n=7,069)

| **Covariate** | **Prevalence (%) (95% CI)** | **Adjusted Odds Ratio, (95% CI)** | **p-value** |
| --- | --- | --- | --- |
| **Overall** | 11.6 (10.8-12.5) | F(22, 6355)=12.73 | **<0.0001** |
| **Age group (years)** |  | F(5,6372)=14.48 | **<0.0001** |
| 16-19 | 17.7 (14.8-21.0) | 2.64 (1.77-3.94) | **<0.0001** |
| 20-24 | 19.4 (17.3-21.8) | 2.97 (2.17-4.06) | **<0.0001** |
| 25-34 | 16.1 (13.9-18.6) | 2.80 (2.04-3.85) | **<0.0001** |
| 35-44 | 11.2 (0.94-13.2) | 2.02 (1.46-2.79) | **<0.0001** |
| 45-54 | 6.1 (4.7-7.9) | 1.08 (0.73-1.58) | 0.713 |
| 55 and older | 5.3 (4.2-6.6) | --ref-- | --ref-- |
| **Sex** |  | F(1,6376)=13.79 | **<0.0001** |
| Female | 10.3 (9.2-11.5) | --ref-- | --ref-- |
| Male | 12.9 (11.7-14.2) | 1.45 (1.19-1.77) | **<0.0001** |
| **Gender modality** |  | F(1,6376)=2.55 | 0.110 |
| Cisgender | 11.5^↑^ (10.7-12.4) | --ref-- | --ref-- |
| Gender diverse | 13.3 (9.9-17.5) | 0.71 (0.47-1.08) | 0.110 |
| **Sexual orientation** |  | F(3, 6374)=0.86 | 0.459 |
| Heterosexual (straight) | 10.8 (9.9-11.7) | --ref-- | --ref-- |
| Homosexual (gay or lesbian) | 15.1 (11.0-20.4) | 1.01 (0.66-1.56) | 0.948 |
| Bisexual or other sexual identity | 16.4 (14.0-19.1) | 0.97 (0.74-1.27) | 0.805 |
| Unstated | 7.2^E^ (4.3-11.8) | 0.57 (0.28-1.14) | 0.110 |
| **Ethnic group** |  | F(2, 6375)=0.80 | 0.448 |
| White (exclusive category) | 10.7 (9.9-11.6) | --ref-- | --ref-- |
| Indigenous | 13.4 (9.9-18.0) | 0.96 (0.62-1.51) | 0.868 |
| Other ethnic group/unstated | 15.1 (13.0-17.6) | 1.17 (0.91-1.50) | 0.215 |
| **Highest education level** |  | F(2, 6375)=2.31 | 0.100 |
| High school or less | 15.7 (14.1-17.4) | 1.30 (1.01-1.67) | 0.039 |
| Trades/college or non-university diploma or certificate | 10.9 (9.4-12.6) | 1.21 (0.95-1.54) | 0.125 |
| At least some university | 9.3 (8.1-10.6) | --ref-- | --ref-- |
|  |  |  |  |
| **Household income** |  | F(2, 6375)=13.72 | **<0.0001** |
| Less than $50,000 | 16.8 (14.7-19.2) | 1.90 (1.49-2.42) | **<0.0001** |
| $50,000-$99,999 | 12.3 (10.8-14.0) | 1.40 (1.13-1.75) | 0.003 |
| $100,000 or more | 8.3 (7.3-9.5) | --ref-- | --ref-- |
| **Mental health status** |  | F(1, 6376)=36.35 | **<0.0001** |
| Fair or Poor | 18.7 (16.9-20.7) | 1.83 (1.50-2.22) | **<0.0001** |
| Other response | 8.9 (8.0-9.8) | --ref-- | --ref-- |
| **Community size** |  | F(2, 6375)=6.83 | **0.001** |
| Rural/small community (<30,000) | 8.9 (7.7-10.3) | --ref-- | --ref-- |
| Medium community (30,000-99,999) | 12.7 (10.8-14.9) | 1.50 (1.15-1.96) | 0.003 |
| Large community (100,000 or more) | 12.7 (11.5-14.0) | 1.49 (1.19-1.86) | **0.001** |
| **Immigration status** |  | F(1, 6376)=0.57 | 0.451 |
| Born outside of Canada | 13.9 (11.4-16.7) | 1.12 (0.84-1.50) | 0.451 |
| Born in Canada | 11.3 (10.4-12.2) | --ref-- | --ref-- |
|  |  |  |  |
| **Provincial/territorial cannabis retail model** |  | F(2, 6375)=3.72 | 0.024 |
| Hybrid (public & hybrid sales) | 11.4 (10.3-12.6) | 1.17 (0.93-1.47) | 0.176 |
| Private sales | 10.5^↑^ (9.0-12.2) | 1.45 (1.11-1.90) | 0.007 |
| Public (government-run) sales | 13.6 (11.8-15.7) | --ref-- | --ref-- |

Significant differences at p<0.003 are bolded.

↑Indicates value should be rounded up if rounded to a whole number.

↓Indicates value should be rounded down if rounded to a whole number.

Ref=reference group

^E^ Estimate should be interpreted with caution due to moderate sampling variability.

Additional Table 9. Felt they needed or received professional help for cannabis use in lifetime, among past 12-month consumers who used cannabis more than once in lifetime, 2023-2024 Canadian Cannabis Survey (n=6,892)

| **Covariate** | **Prevalence (%) (95% CI)** | **Adjusted Odds Ratio, (95% CI)** | **p-value** |
| --- | --- | --- | --- |
| **Overall** | 6.4 (5.7-7.0) | F(19, 6231)=6.96 | **<0.0001** |
| **Age group (years)** |  | F(5, 6227)=6.41 | **<0.0001** |
| 16-19 | 9.7 (7.5-12.5) | 1.98 (1.18-3.32) | 0.010 |
| 20-24 | 10.1 (8.4-11.9) | 2.39 (1.60-3.57) | **<0.0001** |
| 25-34 | 9.6 (7.8-11.7) | 2.71 (1.82-4.03) | **<0.0001** |
| 35-44 | 5.7 (4.4-7.3) | 1.84 (1.22-2.79) | 0.004 |
| 45-54 | 3.6^E^ (2.5-5.2) | 1.20 (0.73-1.98) | 0.477 |
| 55 and older | 2.9 (2.1-3.8) | --ref-- | --ref-- |
| **Sex** |  | F(1, 6231)=9.93 | **0.002** |
| Female | 5.6 (4.7-6.5) | --ref-- | --ref-- |
| Male | 7.1 (6.2-8.1) | 1.52 (1.17-1.98) | **0.002** |
| **Gender modality** |  | F(1, 6231)=0.04 | 0.837 |
| Cisgender | 6.2 (5.6-6.9) | --ref-- | --ref-- |
| Gender diverse | 9.1^E^ (6.2-13.3) | 0.95 (0.56-1.60) | 0.837 |
| **Sexual orientation** |  | F(1, 6231)=0.30 | 0.582 |
| Heterosexual (straight) | 5.7 (5.0-6.4) | --ref-- | --ref-- |
| Other orientation/unstated | 9.1 (7.6-11.0) | 1.09 (0.79-1.51) | 0.582 |
| **Ethnic group** |  | F(1, 6231)=1.32 | 0.251 |
| White (exclusive category) | 5.9 (5.2-6.6) | --ref-- | --ref-- |
| Other ethnic group/unstated | 8.1 (6.6-9.8) | 1.19 (0.88-1.59) | 0.251 |
| **Highest education level** |  | F(2, 6230)=1.65 | 0.192 |
| High school or less | 8.6 (7.3-10.0) | 1.29 (0.94-1.79) | 0.115 |
| Trades/college or non-university diploma or certificate | 6.2 (5.0-7.5) | 1.28 (0.94-1.74) | 0.118 |
| At least some university | 4.8 (4.0-5.9) | --ref-- | --ref-- |
|  |  |  |  |
| **Household income** |  | F(2, 6230)=5.99 | 0.003 |
| Less than $50,000 | 9.6 (7.9-11.5) | 1.75 (1.27-2.41) | **0.001** |
| $50,000-$99,999 | 6.8 (5.7-8.2) | 1.33 (0.99-1.79) | 0.056 |
| $100,000 or more | 4.5 (3.8-5.5) | --ref-- | --ref-- |
| **Mental health status** |  | F(1, 6231)=30.01 | **<0.0001** |
| Fair or Poor | 11.2 (9.7-12.8) | 2.02 (1.57-2.60) | **<0.0001** |
| Other response | 4.5^↓^ (3.9-5.2) | --ref-- | --ref-- |
| **Community size** |  | F(2, 6230)=1.28 | 0.277 |
| Rural/small community (<30,000) | 5.6 (4.6-6.8) | --ref-- | --ref-- |
| Medium community (30,000-99,999) | 6.6 (5.2-8.2) | 1.26 (0.89-1.78) | 0.197 |
| Large community (100,000 or more) | 6.7 (5.8-7.7) | 1.25 (0.93-1.67) | 0.135 |
| **Immigration status** |  | F(1, 6231)=0.01 | 0.925 |
| Born outside of Canada | 6.7 (4.9-9.0) | 0.98 (0.66-1.45) | 0.925 |
| Born in Canada | 6.3 (5.6-7.0) | --ref-- | --ref-- |
|  |  |  |  |
| **Provincial/territorial cannabis retail model** |  | F(2, 6230)=0.33 | 0.716 |
| Hybrid (public & hybrid sales) | 6.2 (5.4-7.1) | 0.87 (0.66-1.19) | 0.428 |
| Private sales | 6.4 (5.2-7.8) | 0.89 (0.63-1.26) | 0.518 |
| Public (government-run) sales | 7.0 (5.7-8.6) | --ref-- | --ref-- |

Significant differences at p<0.003 are bolded.

↑Indicates value should be rounded up if rounded to a whole number.

↓Indicates value should be rounded down if rounded to a whole number.

Ref=reference group

^E^ Estimate should be interpreted with caution due to moderate sampling variability.

Additional Table 10. Often or always combined cannabis with other drug(s) ^a^ in past 12 months, among past 12-month cannabis consumers, 2023-2024 Canadian Cannabis Survey (n=7,238)

| **Covariate** | **Prevalence (%) (95% CI)** | **Adjusted Odds Ratio, (95% CI)** | **p-value** |
| --- | --- | --- | --- |
| **Overall** | 30.0 (28.8-31.2) | F(22, 6512)=10.98 | **<0.0001** |
| **Age group (years)** |  | F(5, 6529)=16.68 | **<0.0001** |
| 16-19 | 43.9 (39.9-48.0) | 2.62 (2.01-3.41) | **<0.0001** |
| 20-24 | 38.7 (35.9-41.4) | 2.32 (1.89-2.83) | **<0.0001** |
| 25-34 | 30.6 (27.7-33.6) | 1.77 (1.44-2.18) | **<0.0001** |
| 35-44 | 31.8 (29.1-34.7) | 1.86 (1.53-2.26) | **<0.0001** |
| 45-54 | 31.1 (28.1-34.2) | 1.82 (1.49-2.24) | **<0.0001** |
| 55 and older | 19.8 (17.8-21.9) | --ref-- | --ref-- |
| **Sex** |  | F(1, 6533)=15.86 | **<0.0001** |
| Female | 27.0 (25.4-28.7) | --ref-- | --ref-- |
| Male | 32.7 (31.1-34.4) | 1.30 (1.14-1.47) | **<0.0001** |
| **Gender modality** |  | F(1, 6533)=1.59 | 0.207 |
| Cisgender | 30.1 (28.9-31.3) | --ref-- | --ref-- |
| Gender diverse | 28.5^↑^ (23.8-33.7) | 0.82 (0.60-1.12) | 0.207 |
| **Sexual orientation** |  | F(3, 6531)=1.71 | 0.163 |
| Heterosexual (straight) | 29.8 (28.5-31.1) | --ref-- | --ref-- |
| Homosexual (lesbian or gay) | 30.4 (24.9-36.5) | 0.84 (0.62-1.14) | 0.260 |
| Bisexual/other sexual identity | 32.3 (29.2-35.6) | 0.86 (0.70-1.06) | 0.167 |
| Unstated | 23.3 (17.3-30.7) | 0.63 (0.38-1.06) | 0.083 |
| **Ethnic group** |  | F(2, 6532)=1.37 | 0.253 |
| White (exclusive category) | 30.0 (28.7-31.4) | --ref-- | --ref-- |
| Indigenous | 36.2 (30.9-42.0) | 1.20 (0.90-1.59) | 0.210 |
| Other ethnic group/unstated | 28.1 (25.4-31.0) | 0.91 (0.76-1.10) | 0.333 |
| **Highest education level** |  | F(2, 6532)=13.32 | **<0.0001** |
| High school or less | 37.5^↓^ (35.3-39.7) | 1.50 (1.27-1.78) | **<0.0001** |
| Trades/college or non-university diploma or certificate | 30.8 (28.6-33.0) | 1.37 (1.18-1.60) | **<0.0001** |
| At least some university | 24.0 (22.3-25.9) | --ref-- | --ref-- |
|  |  |  |  |
| **Household income** |  | F(2, 6532)=2.74 | 0.065 |
| Less than $50,000 | 33.9 (31.2-36.6) | 1.22 (1.03-1.44) | 0.019 |
| $50,000-$99,999 | 29.9 (27.8-32.2) | 1.08 (0.93-1.25) | 0.327 |
| $100,000 or more | 28.3 (26.5-30.1) | --ref-- | --ref-- |
| **Mental health status** |  | F(1, 6533)=11.32 | **0.0008** |
| Fair or Poor | 36.3 (34.0-38.6) | 1.28 (1.10-1.48) | **0.0008** |
| Other response | 27.5^↑^ (26.2-28.9) | --ref-- | --ref-- |
| **Community size** |  | F(2, 6532)=3.33 | 0.036 |
| Rural/small community (<30,000) | 29.9 (27.8-32.1) | --ref-- | --ref-- |
| Medium community (30,000-99,999) | 28.9 (26.3-31.6) | 0.99 (0.83-1.18) | 0.905 |
| Large community (100,000 or more) | 30.5^↓^ (28.8-32.2) | 1.17 (1.02-1.35) | 0.029 |
| **Immigration status** |  | F(1, 6533)=9.06 | 0.003 |
| Born outside of Canada | 22.7 (19.7-25.9) | --ref-- | --ref-- |
| Born in Canada | 31.2 (29.9-32.5) | 1.40 (1.12-1.75) | 0.003 |
|  |  |  |  |
| **Provincial/territorial cannabis retail model** |  | F(2, 6532)=8.08 | **0.0003** |
| Hybrid (public & hybrid sales) | 28.6 (27.1-30.2) | 0.76 (0.66-0.89) | **0.0003** |
| Private sales | 29.3 (27.0-31.8) | 0.72 (0.60-0.86) | **0.0003** |
| Public (government-run) sales | 34.9 (32.4-37.6) | --ref-- | --ref-- |

Significant differences at p<0.003 are bolded.

↑Indicates value should be rounded up if rounded to a whole number.

↓Indicates value should be rounded down if rounded to a whole number.

Ref=reference group

^a^ ‘Combined’ was defined as being mixed or consumed at the same time. Drugs included any of the following: alcohol; tobacco; e-cigarettes with nicotine; opioids; stimulants; sedatives/tranquilizers; and hallucinogens/dissociatives.

Table 11. Used cannabis at/within 2h of work in past 12 months and has a hazardous occupation,^a^ among those employed in the past 12 months, 2023-2024 Canadian Cannabis Survey (n=6,838)

| **Covariate** | **Prevalence (%) (95% CI)** | **Adjusted Odds Ratio, (95% CI)** | **p-value** |
| --- | --- | --- | --- |
| **Overall** | 5.4 (4.8-6.0) | F(19, 6164)=9.02 | **<0.0001** |
| **Age group (years)** |  | F(5, 6178)=2.52 | 0.028 |
| 16-19 | 10.1 (7.9-12.9) | 2.99 (1.32-3.82) | 0.003 |
| 20-24 | 7.5 (6.1-9.1) | 1.97 (1.22-3.16) | 0.005 |
| 25-34 | 5.7 (4.4-7.4) | 2.10 (1.29-3.40) | 0.003 |
| 35-44 | 5.3 (4.2-6.8) | 2.02 (1.26-3.23) | 0.003 |
| 45-54 | 5.0 (3.7-6.7) | 2.05 (1.26-3.35) | 0.004 |
| 55 and older | 2.6^E^ (1.8-3.6) | --ref-- | --ref-- |
| **Sex** |  | F(1, 6182)=30.35 | **<0.0001** |
| Female | 3.8 (3.1-4.6) | --ref-- | --ref-- |
| Male | 6.8 (6.0-7.8) | 2.17 (1.65-2.86) | **<0.0001** |
| **Gender modality** |  | F(1, 6182)=0.31 | 0.578 |
| Cisgender | 5.2 (4.6-5.8) | --ref-- | --ref-- |
| Gender diverse | 8.8^E^ (6.0-12.7) | 1.17 (0.67-2.03) | 0. 578 |
| **Sexual orientation** |  | F(1, 6182)=0.56 | 0.454 |
| Heterosexual (straight) | 5.0 (4.4-5.7) | --ref-- | --ref-- |
| Other orientation/unstated | 6.8 (5.5-8.4) | 1.14 (0.81-1.61) | 0. 454 |
| **Ethnic group** |  | F(1, 6182)=12.34 | **0.0004** |
| White (exclusive category) | 4.8 (4.2-5.4) | --ref-- | --ref-- |
| Other ethnic group/unstated | 7.3 (6.0-8.9) | 1.66 (1.25-2.21) | **0.0004** |
| **Highest education level** |  | F(2, 6181)=18.49 | **<0.0001** |
| High school or less | 9.6 (8.3-11.1) | 3.26 (2.23-4.78) | **<0.0001** |
| Trades/college or non-university diploma or certificate | 5.1 (4.2-6.3) | 2.03 (1.40-2.93) | **<0.0001** |
| At least some university | 2.5^↓^ (1.9-3.2) | --ref-- | --ref-- |
|  |  |  |  |
| **Household income** |  | F(2, 6181)=2.90 | 0.055 |
| Less than $50,000 | 7.0 (5.6-8.6) | 1.44 (1.02-2.02) | 0.038 |
| $50,000-$99,999 | 6.1 (5.1-7.4) | 1.38 (1.02-1.86) | 0.037 |
| $100,000 or more | 3.9 (3.2-4.7) | --ref-- | --ref-- |
| **Mental health status** |  | F(1, 6182)=3.99 | 0.046 |
| Fair or Poor | 7.8 (6.6-9.2) | 1.32 (1.01-1.74) | 0.046 |
| Other response | 4.4 (3.8-5.1) | --ref-- | --ref-- |
| **Community size** |  | F(2, 6181)=0.11 | 0.897 |
| Rural/small community (<30,000) | 5.8 (4.8-7.0) | --ref-- | --ref-- |
| Medium community (30,000-99,999) | 5.6 (4.4-7.2) | 1.02 (0.72-1.46) | 0.898 |
| Large community (100,000 or more) | 4.9 (4.2-5.7) | 0.95 (0.71-1.27) | 0.735 |
| **Immigration status** |  | F(1, 6182)=2.87 | 0.090 |
| Born outside of Canada | 3.9^E^ (2.7-5.6) | 1.48 (0.94-2.32) | 0.090 |
| Born in Canada | 5.6 (5.0-6.3) | --ref-- | --ref-- |
|  |  |  |  |
| **Provincial/territorial cannabis retail model** |  | F(2, 6181)=0.25 | 0.780 |
| Hybrid (public & hybrid sales) | 5.2 (4.4-6.0) | 0.91 (0.67-1.25) | 0.576 |
| Private sales | 5.7 (4.6-7.0) | 0.88 (0.61-1.27) | 0.497 |
| Public (government-run) sales | 5.7 (4.6-7.1) | --ref-- | --ref-- |

Significant differences at p<0.003 are bolded.

↑Indicates value should be rounded up if rounded to a whole number.

↓Indicates value should be rounded down if rounded to a whole number.

Ref=reference group

^E^ Estimate should be interpreted with caution due to moderate sampling variability.

^a^ Hazardous occupations included any of the following: driving a motor vehicle; operating equipment, machinery or tools; working from heights; working with hazardous chemicals, flammable liquids or gases; sharps work; working near hot surfaces, open flames or steam; electrical work; handling loads >20kg; working near flying particles or falling objects; and being responsible for the care/wellbeing of others.

Additional Table 12. Prevalence of reporting ‘usually’ obtaining cannabis from an illicit source (illegal store, illegal website or dealer), among those who have consumed cannabis in the past 12 months, 2023-2024 Canadian Cannabis Survey (n=7,121)

| **Covariate** | **Prevalence (%) (95% CI)** | **Adjusted Odds Ratio, (95% CI)** | **p-value** |
| --- | --- | --- | --- |
| **Overall** | 3.3 (2.9-3.8) | F(15, 6417)=5.51 | **<0.0001** |
| **Age group (years)** |  | F(2,6430)=6.68 | **0.001** |
| 16-19 | 7.2 (5.4-9.7) | --ref-- | --ref-- |
| 20-24 | 2.5^↓^ (1.7-3.5) | 0.36 (0.21-0.62) | **<0.0001** |
| 25 and older | 3.1 (2.6-3.6) | 0.56 (0.35-0.90) | 0.016 |
| **Sex** |  | F(1,6431)=13.32 | **0.0003** |
| Female | 2.5^↓^ (2.0-3.1) | --ref-- | --ref-- |
| Male | 4.1 (3.4-4.9) | 1.83 (1.3-2.5) | **0.0003** |
| **Gender modality** |  | F(1,6431)=0.73 | 0.392 |
| Cisgender | 3.2 (2.8-3.7) | --ref-- | --ref-- |
| Gender diverse | 4.6^E^ (2.8-7.3) | 1.31 (0.71-2.43) | 0.392 |
| **Sexual orientation** |  | F(1,6431)=0.07 | 0.788 |
| Heterosexual (straight) | 3.6 (2.6-4.8) | --ref-- | --ref-- |
| Other sexual orientation | 3.3 (2.8-3.8) | 1.06 (0.70-1.59) | 0.788 |
| **Ethnic group** |  | F(1,6431)=0.07 | 0.791 |
| White (exclusive category) | 3.3 (2.8-3.8) | --ref-- | --ref-- |
| Other ethnic group/unstated | 3.4 (2.5-4.6) | 1.06 (0.70-1.59) | 0.791 |
| **Highest education level** |  | F(2,6430)=2.34 | 0.097 |
| High school or less | 4.7 (3.8-5.8) | 1.54 (1.02-2.33) | 0.041 |
| Trades/college or non-university diploma or certificate | 2.9 (2.2-3.8) | 1.10 (0.73-1.64) | 0.655 |
| At least some university | 2.6 (2.0-3.4) | --ref-- | --ref-- |
|  |  |  |  |
| **Household income** |  | F(2,6430)=2.29 | 0.746 |
| Less than $50,000 | 3.7 (2.8-4.9) | 1.02 (0.68-1.51) | 0.934 |
| $50,000-$99,999 | 3.1 (2.4-4.1) | 0.89 (0.62-1.27) | 0.508 |
| $100,000 or more | 3.3 (2.7-4.1) | --ref-- | --ref-- |
| **Mental health status** |  | F(1,6431)=12.18 | **0.0005** |
| Fair or Poor | 4.7 (3.8-5.9) | 1.75 (1.28-2.40) | **0.0005** |
| Other response | 2.8 (2.3-3.3) | --ref-- | --ref-- |
| **Community size** |  | F(2,6430)=2.04 | 0.131 |
| Rural/small community (<30,000) | 2.9 (2.2-3.7) | --ref-- | --ref-- |
| Medium community (30,000-99,999) | 3.5^↑^ (2.6-4.8) | 1.43 (0.92-2.23) | 0.107 |
| Large community (100,000 or more) | 3.5^↑^ (2.9-4.3) | 1.41 (0.99-2.01) | 0.060 |
| **Immigration status** |  | F(1,6431)=0.40 | 0.527 |
| Born outside of Canada | 3.7^E^ (2.5-5.4) | 1.17 (0.72-1.88) | 0.527 |
| Born in Canada | 3.3 (2.8-3.8) | --ref-- | --ref-- |
|  |  |  |  |
| **Provincial/territorial cannabis retail model** |  | F(1,6431)=10.69 | **0.001** |
| Hybrid/Private sales | 3.0 (2.5-3.5) | --ref-- | --ref-- |
| Public (government-run) sales | 4.7 (3.7-6.1) | 1.70 (1.24-2.34) | **0.001** |

Significant differences at p<0.003 are bolded.

↑Indicates value should be rounded up if rounded to a whole number.

↓Indicates value should be rounded down if rounded to a whole number.

Ref=reference group

^E^ Estimate should be interpreted with caution due to moderate sampling variability.
